# Animal models of maternal high fat diet exposure and effects on metabolism in offspring: a meta‐regression analysis

**DOI:** 10.1111/obr.12524

**Published:** 2017-03-30

**Authors:** G. A. Ribaroff, E. Wastnedge, A. J. Drake, R. M. Sharpe, T. J. G. Chambers

**Affiliations:** ^1^ Edinburgh Medical School, Chancellor's BuildingUniversity of EdinburghEdinburghUK; ^2^ NHS Lothian, University Hospitals DivisionRoyal Hospital for Sick ChildrenEdinburghUK; ^3^ University/BHF Centre for Cardiovascular Science, Queen's Medical Research InstituteUniversity of EdinburghEdinburghUK; ^4^ MRC Centre for Reproductive Health, Queen's Medical Research InstituteUniversity of EdinburghEdinburghUK; ^5^ NHS Lothian, University Hospitals Division, Metabolic UnitWestern General HospitalEdinburghUK

**Keywords:** Adiposity, glucose, lipids, maternal, obesity

## Abstract

Animal models of maternal high fat diet (HFD) demonstrate perturbed offspring metabolism although the effects differ markedly between models. We assessed studies investigating metabolic parameters in the offspring of HFD fed mothers to identify factors explaining these inter‐study differences. A total of 171 papers were identified, which provided data from 6047 offspring. Data were extracted regarding body weight, adiposity, glucose homeostasis and lipidaemia. Information regarding the macronutrient content of diet, species, time point of exposure and gestational weight gain were collected and utilized in meta‐regression models to explore predictive factors. Publication bias was assessed using Egger's regression test.

Maternal HFD exposure did not affect offspring birthweight but increased weaning weight, final bodyweight, adiposity, triglyceridaemia, cholesterolaemia and insulinaemia in both female and male offspring. Hyperglycaemia was found in female offspring only. Meta‐regression analysis identified lactational HFD exposure as a key moderator. The fat content of the diet did not correlate with any outcomes. There was evidence of significant publication bias for all outcomes except birthweight.

Maternal HFD exposure was associated with perturbed metabolism in offspring but between studies was not accounted for by dietary constituents, species, strain or maternal gestational weight gain. Specific weaknesses in experimental design predispose many of the results to bias.

## Introduction

Obesity is a growing public health concern. In 2014, 61.7% of adults and 31.2% of children in the UK were overweight or obese 1. The rising prevalence of obesity is evident amongst pregnant women such that around half of women of childbearing age are now overweight with 5% of mothers giving birth in the UK having a body mass index (BMI) >35 kg m^−2^ 1. This not only increases health risks for the mother but may also have significant impacts on the health and well‐being of offspring with both short and long term sequelae 2, 3, 4. Notably, a number of studies link maternal obesity during pregnancy with an increased risk of obesity in offspring, which persists across the lifespan 5, 6, 7, 8, 9, 10, 11.

Although it is difficult to separate the direct effect of maternal obesity on offspring from other environmental and genetic factors, studies have found that maternal obesity is an independent risk factor for high BMI in her children 7, 9, 11. Furthermore, children born to obese mothers before maternal bariatric surgery are more likely to be obese and to have higher circulating lipids and higher insulin resistance than their siblings born after surgery 12, 13. Being overweight as a child is an independent risk factor for both increased morbidity and premature mortality in adulthood 14. In addition, there is a strong positive association between high childhood BMI and adult obesity 15. From a public health perspective, understanding the interactions between maternal obesity and offspring weight is crucial to inform strategies aimed at ameliorating the growing obesity epidemic.

Epidemiological studies in humans are limited in their ability to assess the independent contribution of maternal obesity to offspring phenotype, as it is difficult to separate the effects of prenatal exposure from a shared post‐natal environment and genetic factors 16. Additionally, the extent to which changes in offspring are a consequence of maternal obesity, rather than of materno‐fetal over nutrition *per se* is unclear 16, although some evidence suggests that a maternal high fat diet (HFD) increases the likelihood of obesity in offspring irrespective of maternal weight 17. Animal models facilitate studies aimed at determining the relative importance of maternal obesity, materno‐fetal over‐nutrition and the postnatal environment; however, significant disparities between studies can lead to difficulties in interpreting results and evaluation of their potential significance for human health.

There are numerous examples of heterogeneity in study methods. In terms of diet, animals may have access to unlimited high‐energy foods or instead be fed a tightly controlled HFD. Diets may be introduced at different stages of development with some studies using experimental diets before pregnancy only and others throughout pregnancy. Furthermore, a variety of animal species and strains have been used. There is no standardized approach to investigations using animal models, and this heterogeneity makes inter‐study comparison and interpretation difficult and introduces multiple uncertainties when attempting to extrapolate findings to humans.

To try to remove such uncertainties, we undertook a systematic review and meta‐regression analysis of existing experimental data. Our aims were to (i) explore the quality of the evidence base; (ii) measure the overall effect of a maternal HFD on the metabolic profile, weight and adiposity of offspring; and (iii) identify factors that might predict offspring adiposity and associated metabolic dysfunction. Using a comprehensive approach for identifying determinants of metabolic outcomes in animal models of maternal HFD exposure, this review helps to identify factors that may be important in a human context.

## Methods

### Literature search and study selection

In order to identify studies in which offspring outcomes had been assessed following maternal HFD exposure, we searched MEDLINE, CAB and EMBASE using the search strategy shown in Table 1. An alternative search strategy was used for Web of Science (which does not use controlled vocabulary): (Offspring OR daughter OR daughters OR son OR sons) AND (HFD OR ‘high fat’ OR ‘induced obesity’) AND (lactation or gestation* or maternal or ‘developmental programming’ or ‘fetal programming’). The search was conducted on 6 July 2015. Studies were identified from the earliest year available in each database; MEDLINE 1946, CAB 1973, and EMBASE 1980 and Web of Science back to 1900. Duplicate abstracts were identified and removed using Mendeley (Elsevier, US). Abstracts and titles were reviewed by TC, GR and EW, identifying all studies in which outcomes in offspring were measured following HFD exposure pre‐pregnancy or during pregnancy or lactation. We excluded human studies, studies not published in English and those in which offspring were re‐challenged with a HFD, but where the control arm continued on the control diet making comparisons difficult; 10% of the abstracts, selected sequentially, were rechecked by a second reviewer. Any queries were resolved by discussion between the three reviewers. Conference abstracts were excluded, but a further database search of the authors and keywords from the abstract was undertaken to check for any publications of these results. Full text papers were accessed for the identified abstracts. Study quality and data extraction were performed using the same form. All data were extracted by TC, GR and EW.

**Table 1:** Search strategy. The following terms were included in the search for MEDLINE, CAB and EMBASE. The following terms were included in the search including the logical terms in rows 15–18

ID	Search terms
2	Prenatal Exposure Delayed Effects/
3	exp Peripartum Period/
4	offspring.ti,ab.
5	daughter*.ti,ab.
6	son*.ti,ab.
7	lactation.ti,ab.
8	gestation*.ti,ab.
9	maternal.ti,ab.
10	exp Fats/
11	exp Diet, High‐Fat/
12	hfd.ti,ab.
13	‘induced obesity’.ti,ab.
14	‘high fat’.ti,ab.
15	11 or 12 or 13 or 14 or 15
16	1 or 2 or 3 or 4 or 8 or 9 or 10
17	5 or 6 or 7
18	16 and 17 and 18

exp, expansion of MESH term; ti, title; ab, abstract.

To evaluate the quality of evidence, papers were screened for the adequacy of the control group, statement of the number of animals used, if the intervention group was randomly selected and if statistical account was taken of animals from the same litter not having true independence (by ‘nesting’ or use of 1 animal per litter). We also recorded if researchers were blinded to the treatment groups of animals. Each of these methodological design factors can bias interpretation of results.

We aimed to identify factors responsible for heterogeneity between studies. To this end, we hypothesized that the following factors might influence study results: (i) the species and strain of animals investigated; (ii) the specific intervention and control diets used and the extent to which the diets were matched for micronutrient and macronutrient content; (iii) the specific methods used for assessment of glycaemia, insulin resistance and adiposity; and (iv) the effects of the intervention diet on the mother, e.g. gestational weight gain.

Thus, to identify potential predictive factors, details regarding the control and intervention diets were extracted, including if a ‘cafeteria’ style diet was used. We also checked for control of micronutrient consumption (use of ‘matched’ control and intervention diets). Fat, protein and carbohydrate content as a proportion of Kcal was recorded, and if not stated these were determined by accessing data from the diet manufacturers, or by using absolute content/kg of each macronutrient to calculate macronutrient content as a proportion of total Kcal. The age of the females at the start of the intervention was recorded in days, along with the length of time of exposure before mating. Exposure during the first, second and third third of total gestation (in rodents equating first, second and third weeks) was recorded together with whether this continued into lactation. Where data were available for exposure during gestation and lactation (for example in a cross‐fostering model), we extracted just the data from the control arm and from the arm exposed to HFD during both gestation and lactation in order to reduce the risk of ‘double counting’ subjects in the meta‐analysis.

Experimental methods were recorded. For adiposity: weight of fat pads using the first anatomical region from the following order: (gonadal, perirenal, omental), nuclear magnetic resonance or dual energy X‐ray absorptiometry. For glucose levels: non‐fasting, fasting, oral glucose tolerance test and intraperitoneal glucose tolerance test. For insulin levels: non‐fasting, fasting and insulin tolerance test. Information about housing (single/number of animals per cage) was recorded. Finally, pre‐breeding and post lactation maternal bodyweight, adiposity, glucose, insulin, triglycerides, cholesterol and leptin results were recorded (if presented) in order to try to ascertain if the maternal metabolic response to the diet was an important factor in predicting outcomes in offspring. Most studies cull litters to specific sizes in order to try and remove bias introduced by difference in litter sizes. Where this occurred, it was recorded, along with the number of animals kept in each litter.

In order to compare studies that exhibited variability in methodology, we collected information to enable calculation of standardized mean differences (SMD) (analogous to Hedges' *g*). Thus, where available, we collected the mean and SD (or SEM) of birthweight, weaning weights (at ~ postnatal day 21) and endpoint weight, adiposity, glucose, insulin, triglycerides, cholesterol and leptin for sons and daughters or grouped (i.e. compiled male and female) offspring. Further studies were excluded at this point if (i) non‐parametric data were presented as we would be unable to incorporate these into the analysis, or (ii) if none of the outcome measures collected were presented in the study. *Plotdigitizer* (http://plotdigitizer.sourceforge.net/) was used to extract data from graphical figures. Where details from the studies were unclear (for example the *N* of the presented data), corresponding authors were contacted for verification.

### Data analysis

Data were entered into an excel spreadsheet (available as a supplementary file); 10% of studies were selected at random for validation of data extraction and data input by GR and EW. Data were imported into R version 3.2.3, and the metafor package v1.9‐8 was used for meta‐regression analysis 18. To allow comparison between different studies, SMD was calculated for each outcome using the *escalc* function.

A basic meta‐analysis was conducted using the *rma* function. We used a random effects model fitted with restricted maximum‐likelihood estimator to allow for inter‐ and intra‐study variation; 95% confidence intervals were predicted. Given the high intra‐study heterogeneity (assessed by *I* ^2^ values >50%), further meta‐regression was conducted to try to identify predictors. The factors identified *a priori*, which we predicted might influence outcome, were added individually to the random effects model as moderators, again using the *rma* function.

To account for possible effects of macronutrient differences, we first excluded studies using a cafeteria‐style diet (as no macronutrient data were available for the intervention arm); then the ratio of fat, carbohydrate and protein between the HFD and CD diets was calculated. This ratio was included into the random effects model as a continuous moderator. In a similar manner, the fold change in maternal weight pre‐ and post‐dietary exposure (where presented) was calculated and examined as a continuous moderator. Thus, the following analyses were run: random assignment, use of nesting or one animal/litter, use of cafeteria diet, use of matched diets and exposure during early, mid or late gestation or during lactation, species and strain, each as discrete variables and as continuous predictors, fat, carbohydrate and protein ratio, length of exposure pre‐mating and age at final outcome measures.

Data are presented as estimated SMD and 95% confidence intervals in forest plots drawn using the *forestplot* package with the basic meta‐analysis presented above the meta‐regression models. Publication bias was identified with Egger's regression tests and visualized using funnel plots.

## Results

The literature searches identified 5144 abstracts, and 1844 remained following removal of duplicates. Of these, 447 were studies in which maternal diet was manipulated and the outcomes of interest were examined in offspring; 192 of these were conference abstracts leaving 255 papers for review. A further 84 of these were excluded following review of the paper because postnatal outcomes were not assessed (*n* = 43), there were methodological problems (*n* = 21), the paper was not available in English (*n* = 4), non‐parametric data were observed (*n* = 5), or insufficient information was available to determine the SMD and no response was received from the corresponding authors (*n* = 10). Furthermore, we were unable to obtain the manuscript for one paper from the British Library. This left 171 studies included in the quantitative meta‐analysis (Fig. S1). We were able to pool data collected from over 1400 females exposed to a HFD peri‐gestation and lactation, which gave birth to a total of 6047 offspring included in the meta‐analysis.

### Birthweight

**Figure 1:**
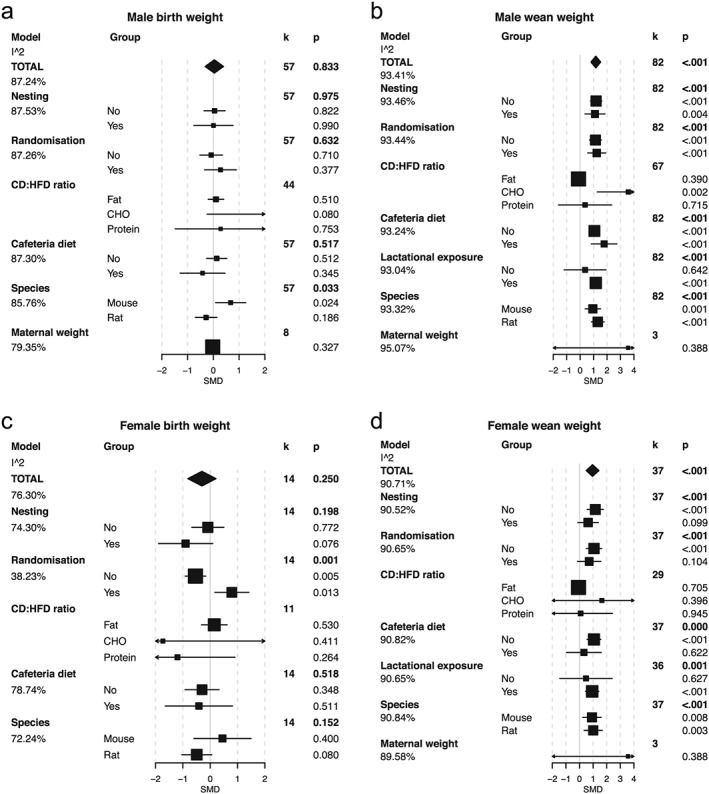
Meta‐analysis and meta‐regression of birth weight and wean weight in the male and female offspring of mothers exposed to a high fat diet (HFD) during gestation and lactation. a. Male birth weight, b. male wean weight, c. female birthweight, d. female wean weight. In the TOTAL model, estimated standardized mean differences (SMD) and 95% confidence intervals are presented as a summary of all studies. *k* refers to the number of studies included. The significance of the effect size was assessed by random‐effects model analysis. Explanation for heterogeneity was explored by meta‐regression by including various moderating factors into the random‐effects model. These included the following: *nesting* – the use of statistical procedures to account for non‐independence of animals from the same litter; *randomization* – the random assignment of animals to each intervention group; *CD:HFD ratio* of macronutrients, fat, carbohydrate (CHO) and protein; *cafeteria diet* – the use of choice diet or supplementation of standard diets with palatable energy‐rich foods; *species*; and *maternal weight* – an approximation for gestation weight gain taken as the ratio change in weight from pre mating to post lactation. Estimates for the SMD and 95% confidence intervals are presented for these models along with the residual heterogeneity unaccounted for in the model (the I^2 beneath each model).

Birthweight data were obtained from 57 studies for male and 14 studies for female offspring following maternal HFD exposure. Maternal dietary exposure had no overall effect on birthweight (Fig. 1a,c). However, where species was taken into account, maternal HFD exposure increased birthweight in male mice, and there was a trend in the opposite direction in male rats (Fig. 1a). The same trend for increased birthweight in mice and reduced birthweight in rats was also observed in the females although this did not reach statistical significance (Fig. 1c). No significant effect was found in the 14 studies reporting combined male and female offspring data (Fig. S3). We found no evidence for publication bias in the male birthweight data with Egger's test (Table S1); however, the female data exhibited a skew suggestive of publication bias (Egger's test *p* = 0.008) (Table S1).

### Bodyweight at weaning

Maternal HFD exposure was associated with increased bodyweight at weaning in both male and female offspring (Fig. 1b,d). There was strong heterogeneity in both male and female data (*I* ^2^ > 90%), which was not accounted for by any of the moderating factors assessed. Interestingly, in studies selected based upon dietary fat content, carbohydrate content was the only macronutrient predictive of this increase in weight (significantly in the male offspring) (Fig. 1b). In the male offspring, a higher proportion of carbohydrate in the control and intervention diets was associated with increasing weaning weight (Spearman's rank *r* = 0.3, *p* = 0.01; Fig. S2a). Exposure to the intervention diet during lactation was a strong predictor for this response in both male and female offspring (Fig. 1b,d). The use of randomization and ‘nesting’ in the experimental design was associated with a diminished effect on weaning bodyweight. Use of the cafeteria diet also diminished the effect on weaning bodyweight observed in the female offspring. Both male and female weaning bodyweight showed evidence of significant publication bias (Fig. S5 and Table S1).

### Final (adult) bodyweight

**Figure 2:**
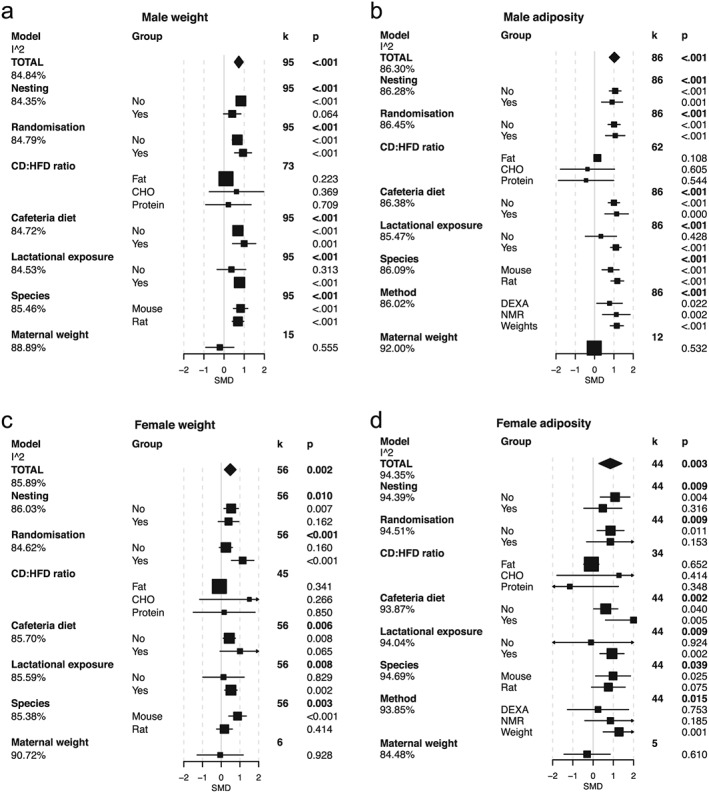
Meta‐analysis and meta‐regression of final body weight and adiposity in the male and female offspring of mothers exposed to a high fat diet (HFD) during gestation and lactation. a. Male body weight, b. male adiposity, c. female body weight d. female adiposity. In the TOTAL model, estimated standardized mean differences (SMD) and 95% confidence intervals are presented as a summary of all studies. *k* refers to the number of studies included. The significance of the effect size was assessed by random‐effects model analysis. Intra study heterogeneity (as a percentage) is shown as the I^2 value. Explanation for heterogeneity was explored by meta‐regression by including various moderating factors into the random‐effects model. These included the following: *nesting* – the use of statistical procedures to account for non‐independence of animals from the same litter; *randomization* – the random assignment of animals to each intervention group; *CD:HFD ratio* of macronutrients, fat, carbohydrate (CHO) and protein; *cafeteria diet* – the use of choice diet or supplementation of standard diets with palatable energy‐rich foods; *species*; the *method* by which adiposity was assessed, fat pad weights, dual energy X‐ray absorptiometry (DEXA) scan or nuclear magnetic resonance (NMR) scan; and *maternal weight* – an approximation for gestation weight gain taken as the ratio change in weight from pre mating to post lactation. Estimates for the SMD and 95% confidence intervals are presented for these models along with the residual heterogeneity unaccounted for in the model (the I^2 beneath each model).

**Figure 3:**
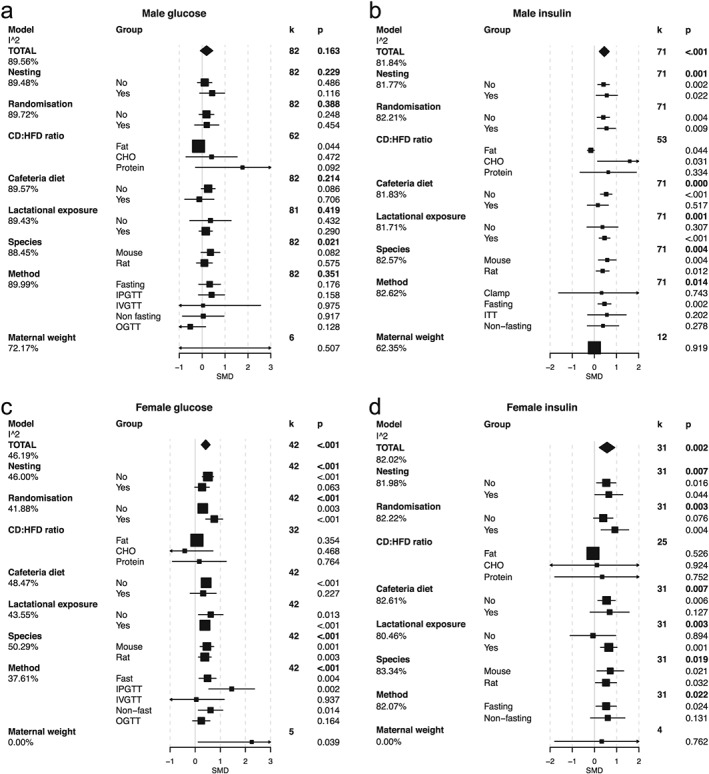
Meta‐analysis and meta‐regression of glucose homeostasis in the male and female offspring of mothers exposed to a high fat diet (HFD) during gestation and lactation. a. Male glucose, b. male insulin, c. female glucose, d. female insulin. In the TOTAL model, estimated standardized mean differences (SMD) and 95% confidence intervals are presented as a summary of all studies. *k* refers to the number of studies included. The significance of the effect size was assessed by random‐effects model analysis. Intra study heterogeneity (as a percentage) is shown as the I^2 value. Explanation for heterogeneity was explored by meta‐regression by including various moderating factors into the random‐effects model. These included the following: *nesting* – the use of statistical procedures to account for non‐independence of animals from the same litter; *randomization* – the random assignment of animals to each intervention group; *CD:HFD ratio* of macronutrients, fat, carbohydrate (CHO) and protein; *cafeteria diet* – the use of choice diet or supplementation of standard diets with palatable energy‐rich foods; *species*; the *method* by which glucose–insulin homeostasis was assessed; and *maternal weight* – an approximation for gestation weight gain taken as the ratio change in weight from pre mating to post lactation. Estimates for the SMD and 95% confidence intervals are presented for these models along with the residual heterogeneity unaccounted for in the model (the I^2 beneath each model).

Male and female final bodyweight was significantly increased following maternal exposure to HFD (Fig. 2a,c). In the male offspring, this effect was independent of the use of randomization or taking account of litters in the statistical analysis. Again, as with the weaning weights, exposure to the intervention diet during lactation was an important determinant of this response. Increased final bodyweight was independent of species used in male offspring but in females was limited to studies in mice, with no significant effect demonstrated in female rat offspring. Seven studies combined male and female offspring data, and in these no effect on adult bodyweight was observed (Fig. S3e). Again, there was strong evidence for publication bias (Figs 3f and Table S1).

### Adiposity

Data regarding adiposity were extracted from 86 studies reporting male outcomes and 44 studies reporting those in females. Here, we included data from weight of fat pads and adiposity determined by dual energy X‐ray absorptiometry or nuclear magnetic resonance. Corresponding to the increased bodyweight, adiposity was increased in both male and female offspring of mothers fed a HFD (Fig. 2b,d). In male offspring, this finding was independent of the use of randomization of the mother to diet, use of nesting in analysis, use of cafeteria diet, species or the method used to determine adiposity (Fig. 2b). However, in the female offspring, the effect on adiposity was more variable and was lost in studies that used randomization or nesting, and the effect was only significant in mice, not in rats. The findings on adiposity matched those for leptin concentrations (Fig. S4a,b). For both adiposity and leptin concentrations, there was strong intra‐study heterogeneity in the results, which was not accounted for by any of the factors included in our analyses, and results appear to be strongly influenced by publication bias (Fig. S5o–r and Table S1).

### Lipids

In the general meta‐analyses, there was a significant increase in triglycerides and cholesterol levels in both male and female offspring of females exposed to HFD (Fig. 3a–d). This effect was clearer in mice than in rats and was more pronounced in studies in which HFD exposure extended beyond gestation into the lactational period. The heterogeneity of study results was not explained by macronutrient content of the diets, or use of nesting or randomization in the experimental design, although female offspring lipid profiles showed evidence of influence by proportion of carbohydrate. Taking into account maternal weight gain reduced much of the heterogeneity in the male offspring triglyceride results (Fig. 3a–d). The male offspring lipid data showed strong evidence for publication bias, which was also apparent in the lipid data for female offspring although with reduced significance (Fig. S5k–n and Table S1).

### Glucose

**Figure 4:**
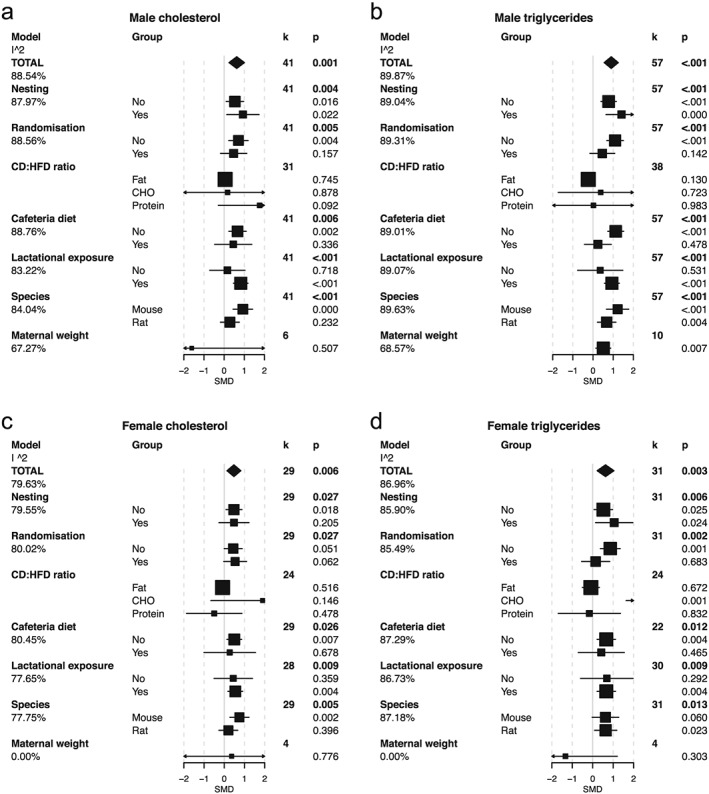
Meta‐analysis and meta‐regression of lipidaemia in the male and female offspring of mothers exposed to a high fat diet (HFD) during gestation and lactation. a. Male cholesterol, b. male triglycerides, c. female cholesterol, d. female triglycerides. In the TOTAL model, estimated standardized mean differences (SMD) and 95% confidence intervals are presented as a summary of all studies. *k* refers to the number of studies included. The significance of the effect size was assessed by random‐effects model analysis. Intra study heterogeneity (as a percentage) is shown as the I^2 value. Explanation for heterogeneity was explored by meta‐regression by including various moderating factors into the random‐effects model. These included the following: *nesting* – the use of statistical procedures to account for non‐independence of animals from the same litter; *randomization* – the random assignment of animals to each intervention group; *CD:HFD ratio* of macronutrients, fat, carbohydrate (CHO) and protein; *cafeteria diet* – the use of choice diet or supplementation of standard diets with palatable energy‐rich foods; *species*; and *maternal weight* – an approximation for gestation weight gain taken as the ratio change in weight from pre mating to post lactation. Estimates for the SMD and 95% confidence intervals are presented for these models along with the residual heterogeneity unaccounted for in the model (the I^2 beneath each model).

Data were extracted from 82 studies regarding glucose homeostasis in male offspring There was no effect of maternal HFD exposure on glucose levels in male offspring (Fig. 4a). In contrast, in the 42 studies involving female offspring, maternal HFD exposure was associated with hyperglycaemia. Data for female offspring glycaemia had lower heterogeneity than in other analyses (*I* ^2^ = 46%). Some of this heterogeneity was further explained by method, with the clearest effects being found in fasting and IPGTT studies (Fig. 4c). There was no evidence for publication bias for the male offspring data, in which no overall change was found. The female offspring data, however, were suggestive of publication bias (Fig. S5f,g and Table S1).

### Insulin

We obtained data from 71 (male) and 31 (female) studies that examined the effect of maternal HFD on offspring insulin concentrations. These were assessed either in fasting or non‐fasting states or during insulin tolerance tests. Although the readouts from these tests have slightly different physiological interpretations, higher insulin concentrations in either test suggest insulin resistance, and the use of SMD helps to compare relative rather than absolute changes in these parameters. Insulin concentrations were higher in both male and female offspring of mothers consuming a HFD (Fig. 4b,d). This effect was dependent upon HFD exposure including the lactational period and was mainly apparent in the fasting insulin concentrations. The effect of maternal HFD was independent of species. None of the factors explored were able to explain the heterogeneity of the results. Again, there was evidence of publication bias (Fig. S5h,i and Table S1).

## Discussion

Here, we present a comprehensive meta‐analysis of experimental models of maternal HFD exposure on metabolic parameters in offspring. Maternal exposure to HFD results in increased bodyweight at weaning and in adulthood along with increased adiposity, blood lipid levels and hyperinsulinaemia in both male and female offspring. We found significantly increased glucose concentrations only in female offspring of HFD‐fed mothers and no effect upon birthweight in either male or female offspring. Our meta‐analysis exposed considerable heterogeneity between studies. Accordingly, we conducted meta‐regression analyses based upon predefined factors that we predicted might be responsible for the inter‐study heterogeneity. These included analysis strategy (use of nesting or one animal per litter), the effect of randomizing females to receive the HFD, effects of relative protein, carbohydrate and fat levels between the control and HFD, use of a cafeteria style diet, exposure during lactation (as well as gestation), the species used and the relative maternal weight gain whilst consuming the HFD. On the whole, these factors did not explain the heterogeneity of the findings, although we did find significant associations between some of the factors and the outcome data presented. These are discussed in more details below. Finally, we found evidence for publication bias in most of the outcomes assessed, suggesting that studies with ‘negative’ findings were less likely to have been published.

### Nesting

A key factor to be considered in these studies is that of multiparous births. In order for the conclusions to be valid, it is necessary that litters are accounted for, such that each exposed female counts as one experimental unit. The use of each individual offspring as an experimental unit can give rise to type 1 errors 19, 20. In order to explore this in our meta‐analysis, we identified studies accounting for litter (either using one animal randomly selected per litter, a litter mean or by including litter as a factor in mixed model analysis of the data). Where no reference was made to account for litter in a study, it was assumed that none had been taken. Only 22% of the papers included in the meta‐analysis accounted for the litter effect clearly in their methods. As would be expected, accounting for the litter reduced the effect size in the meta‐regression analysis. However, an effect of maternal HFD exposure remained significant for male offspring weaning bodyweight, adiposity, lipid and insulin concentrations, and for female offspring triglyceride and insulin concentrations. Thus, we conclude that maternal HFD exposure likely affects metabolic parameters in offspring, although the size of the effect is likely to be overestimated in the current literature and retains a risk of type I statistical error.

### Randomization

Good experimental design stipulates that treatments should be assigned such that each experimental unit has a known chance of receiving a given treatment. Assigning each individual in this way (randomization) is critical as there are often sources of bias, known or unknown, which may influence results. Reducing bias will improve the reliability and interpretability of results 21, 22. Only 31% of the papers from which we extracted data explicitly stated that subjects were randomly allocated to a treatment group. Further bias in studies can be removed by blinding investigators to treatments. None of the studies included in the analysis stated that investigators were blinded. Use of randomization in studies reduced the effect size of outcomes noted in the offspring of HFD‐exposed mothers. When accounting for randomization, significant effects of maternal HFD were still observed on male offspring weaning bodyweight and adiposity and on female offspring insulin and glucose concentrations. These findings highlight the importance of good experimental design and highlight the risk of bias in the studies included in our meta‐analysis.

### Macronutrients

In a meta‐analysis of studies selected for the relative fat content of diets, we unexpectedly found no direct evidence that increased maternal fat consumption increased the occurrence of metabolic abnormalities in the offspring. We assessed relative as opposed to absolute fat content as there was strong heterogeneity between the control diets used in the studies. Whilst no linear relationship was found between the ratio of fat in the control and HFDs and any of the metabolic outcomes assessed, the carbohydrate content of diets was predictive of weaning weight in male offspring (Fig. S2a). However, this was an isolated finding, and there was no significant effect of carbohydrate in the other outcomes assessed. Nevertheless, this finding is consistent with a human randomized controlled trial in which mothers received a hypocaloric Mediterranean style diet versus their usual diet, which found that maternal consumption of carbohydrate in the last weeks of gestation was strongly associated with neonatal body composition, a finding that was independent of maternal BMI and gestational weight gain 23.

Metabolic outcomes in the offspring may thus have been influenced by a number of downstream effects of the HFD in mothers. For example, changes in glucose homeostasis in mothers during pregnancy and lactation, the impact of gestational weight gain and the endocrine changes associated with maternal obesity. In our analysis, we did not find any clear link between gestational weight gain and the offspring outcomes assessed. We collected data regarding maternal glucose homeostasis, adiposity, lipids and leptin levels, but there were insufficient reports from too heterogeneous a population (in terms of time point) for us to draw any meaningful conclusions as to potential influencing factors.

Although the results of this analysis do not address potential mechanisms resulting in adverse metabolic outcomes in offspring of HFD‐fed mothers, they do suggest that a direct effect of maternal dietary fat intake is unlikely, a finding of relevance to the current debate on the optimal diet in humans. There is also evidence that it is not associated with appetite; a recent meta‐analysis showed a maternal obesogenic diet during pregnancy was associated with increased weight gain in offspring that was independent of the quantity of food consumed 24.

### Dietary constituents

In the cafeteria diet paradigm, animals are allowed free access to highly palatable energy dense foods, which promotes hyperphagia and rapid weight gain. Although this reliably induces a metabolic syndrome type picture in exposed animals, it is impossible to control for the effects of individual dietary constituents 25. Alternatively, diet‐induced obesity models include the simple exchange of carbohydrate‐derived calories for fat‐derived calories in comparison to non‐matched chow diets, or experimental ‘matched’ control diets, which attempt to control macro‐ and micro‐nutrient consumption 25. Experimentally, these matched diets allow for more variables to be accounted for, although they do not accurately model the nutrition of Western human society. Our analysis found maternal cafeteria‐style diets led to greater offspring body weight, whereas diet‐induced obesity was associated with increased offspring lipids and insulin concentrations. This might suggest different mechanisms for the programming of these metabolic phenotypes, potentially explained by differences in the caloric or nutritional makeups of these diets 25, 26.

### Gestational and lactational exposure

We included studies in which exposure to the obesogenic diet occurred pre‐pregnancy, during pregnancy and during lactation. We analysed whether the stage of gestation of HFD exposure had any impact upon outcomes in offspring but found no association (data not shown). There was however a strong link between all of the outcomes and exposure to HFD during lactation along with gestation. These effects lasted long after weaning had been completed.

Twenty‐five studies included an experimental design in which cross‐fostering was employed and offspring were switched to a mother that had been fed on the HFD or the CD during gestation. In order to avoid counting the control groups in these studies twice, we selected only animals exposed to HFD *in utero* and during lactation. Thus, there is potential for bias in the presented results with an under‐representation of studies of animals exposed in gestation but not in lactation. Review of the studies employing cross‐fostering indicates general consistency with our findings and suggests that lactational HFD exposure is critical for metabolic changes to occur in the offspring: HFD exposure during lactation was found to be an independent predictor of increased offspring body fat 27, 28, 29, 30 and had a significantly greater effect on offspring body fat than did HFD exposure during pregnancy 27, 28, 30. High fat diet exposure during lactation was also associated with hyperinsulinaemia and hyperglycaemia in offspring 27, 30. This is consistent with the findings of the meta‐regression analysis by Lagisz *et al.* in which offspring bodyweight was increased, especially when HFD exposure continued into lactation 24. Given that rodents are born at a more immature stage than humans, HFD exposure during lactation in rodents might be comparable to that during the last trimester in humans. These data match the human data presented by Renault *et al.* in which diet late in gestation was associated with the biggest changes in neonatal body composition 23.

### Species

The strain of laboratory animals has a significant impact on the susceptibility to development of diabetes and the ‘metabolic syndrome’ 31, 32, 33. We found in general that mouse strains exhibited a stronger offspring metabolic phenotype than did rats after maternal HFD exposure. Whilst differences between species are often suggested as a reason to explain heterogeneity between studies, we found that it accounted for very little of the variability observed in our analysis. We also analysed the strain of animal and found the strongest phenotypes observed in C57bl6 mice and Sprague Dawley rats (data not shown), although again, little of the heterogeneity between studies was accounted for by strain. Results were also included from a small number of studies in other species (macaque, pig and rabbit); however, their numbers were insufficient to be interpretable in the meta‐regression.

### Maternal weight gain

We were unable to identify a relationship between gestational weight gain of HFD‐fed mothers and offspring phenotype in our analysis. However, data regarding gestational weight gain was largely absent from the studies from which data were extracted, resulting in a smaller number of studies available for analysis. However, we did find an association between maternal weight gain and male offspring triglyceride levels. This was consistent with the findings of the Jerusalem perinatal outcomes study, in which gestational weight gain was associated with increased triglyceride levels at age 32, although this association was largely accounted for by BMI at this time point 34. In this human study, which is the largest prospective study with adult outcomes available to date, waist circumference and systolic blood pressure were also associated with gestational weight gain, but these outcomes were also accounted for by offspring BMI as adults. A large Danish cohort study also found that there was a significant independent association with weight gain during pregnancy and high BMI in offspring but only in offspring of overweight and obese mothers – there was no association in offspring from normal weight mothers 35. In humans, childhood obesity has been shown to be independently associated with maternal gestational weight gain, but we were unable to confirm this in the animal studies for which data were available 36.

### Sex‐specific effects

A further important finding of the meta‐analysis was the impact of offspring sex on metabolic outcomes. The clearest of these was that female offspring of HFD‐exposed mothers were hyperglycaemic, whereas male offspring developed hyperinsulinaemia in the absence of hyperglycaemia. Sexual dimorphism in metabolic outcomes is a common finding in the literature and was seen in a number of the studies included in the meta‐analysis. Mirroring our findings, a cross‐sectional linkage human study (in which offspring were not particularly well matched) found that maternal diabetes was associated with increased glucose levels in female but not male human offspring 37.

A potential mechanism here lies in the placenta, in which some studies have found different responses to maternal hormones and diet depending on the sex of the offspring 38, 39, 40.

From an evolutionary perspective, there is some suggestion that sexual dimorphism in metabolism, which preferentially favours the development of female offspring, has developed because female offspring need to be able to withstand greater physiological insult in order to maintain the survival of the species. Female physiology needs to be optimized to withstand pregnancy and lactation, whereas males only need to be able to produce germ cells and mate 39. Therefore, it could be argued that it is more important for female offspring to functionally adapt to sub‐optimal environmental conditions in order to maximize the overall chance of species survival 39. However, our findings do not largely support this hypothesis given that the largest effect sizes were generally observed in the male offspring. This clear sexual dimorphism highlights not only the importance of testing both male and female offspring but also avoiding analysing both sexes together.

### Origin of animals

As we had collected data regarding the origin of animals used in experiments, it was possible to run analyses for all of the outcomes against origin. Interestingly, this appeared to account for some of the differences, in particular male adiposity, weight and insulinaemia. This is obviously confounded by different laboratory groups tending to obtain animals from the same supplier, and the same strain tending to originate from the same supplier, and thus this analysis data is not presented.

### Human relevance

One of the key issues with animal studies is their applicability to humans. Although a number of significant effects of maternal diet on offspring metabolism have been shown, the extent to which these can be extrapolated to humans is uncertain. Whilst there are a number of cohort‐based human studies looking at the impact of maternal obesity on offspring phenotype, the ethics of introducing dietary interventions mean it is difficult to tease out the independent effects of maternal obesity, maternal diet and environmental factors on offspring. There is evidence, however, that gestational diet in humans can independently affect offspring metabolism. One such study showed that high carbohydrate diet during pregnancy leads to greater offspring adiposity 23. A randomized control trial also found that a low glycemic index diet during pregnancy resulted in lower infant adiposity at six months of age 41. High fat diet during pregnancy is also associated with increased offspring adiposity in two randomized control trials 42, 43. Gestational weight gain also appears to be important. One large Danish trial showed that if women followed dietary advice during pregnancy, infants were less likely to be born with high birth weight 3. It appears therefore that the available data for human outcomes seem to correlate with the animal study data shown. A key exception is that of birthweight, which was not significantly affected in the animal meta‐analysis but which in humans is increased in the offspring of obese mothers 44.

### Unaccounted heterogeneity

We predicted that factors such as species, makeup of diet and timing of exposure might help to predict the likelihood of maternal diet influencing offspring phenotype. However, surprisingly, in our analyses, these moderating factors were only able to account for a small amount of the heterogeneity between studies. We thus are unable to explain much of the variation observed between studies. Further factors about which information is less routinely included in the methods sections of papers may be of importance in determining effects, for example the acclimatization and derivation of animals or variation in the sires used. It may also be that the maternal metabolic response to the HFD is indicative of changes in offspring but as maternal phenotyping was not presented in a sufficiently homogenous way, we were unable to assess its importance in our analysis. Publishing such details with future studies might help to identify if any of these factors are of genuine importance.

## Conclusions

We present a comprehensive analysis of animal studies that have investigated the impact of maternal obesogenic diet exposure on offspring metabolism. We demonstrate that although this exposure did not affect birthweight, it was associated with increased wean weight, final bodyweight, adiposity, hypertriglyceridaemia, hypercholesterolaemia and hyperinsulinaemia in female and male offspring, and with hyperglycaemia in female offspring only. We identified specific weaknesses in experimental design, predisposing many of the results to bias. In particular, the use of randomization, experimenter blinding and statistical analysis to take account of litters would improve the robustness of results of future studies. We also found strong evidence for publication bias in the majority of outcomes, which suggests that the effect sizes identified are likely to be over‐estimates.

Although we consistently found that maternal HFD exposure led to perturbed metabolic phenotype in offspring, the results demonstrated marked heterogeneity, which we were unable to account for by taking into account the makeup of diet, species, strain or maternal gestational weight gain. The fact that the fat content of the HFD diets did not appear to correlate with the metabolic changes in offspring suggests the outcomes observed are unlikely to be a direct result of fat exposure. There was a weak association between carbohydrate content of diets and offspring outcomes and more robust evidence that exposure to HFD during lactation was strongly predictive of perturbed offspring metabolism. Thus, taken together (and consistent with human data), the data suggest that a focus of future studies should be maternal carbohydrate consumption during late gestation and the immediate post‐natal (lactational) period.

We hope the information presented here helps to inform future experimental design to elucidate mechanisms responsible for the increased susceptibly in humans to obesity and cardio‐metabolic risk as a result of peri‐gestational maternal diet.

## Conflict of interest statement

No conflict of interest was declared.

## Supporting information


**Figure S1:** PRISMA chart demonstrating the process for selection of articles.

**Figure S2:** Correlations between (a) carbohydrate and (b) fat ratio between intervention and control diets and weaning weight in offspring. Points indicate individual studies. The size of the point is proportional to the inverse square root of the variance in the standardized mean difference of wean weight calculated for each study. A linear model for the fit between the macronutrient ratio and the weaning weight is indicated on each graph. In (a), the correlation between the ratio of carbohydrate content of the diets and wean weight was significant when assessed by Spearman's rank. There was no significant correlation between fat content ratio and wean weight in male offspring.

**Figure S3:** Metabolic outcomes in the offspring of females exposed to HFD: forest plots from studies where male and female data combined. (a) Birthweight, (b) glucose, (c) weaning weight, (d) insulin, (e) final body weight, (f) adiposity, (g) cholesterol, (h) triglycerides, (i) leptin. In the TOTAL model, estimated SMD and 95% confidence intervals are presented as a summary of all studies. *k* refers to the number of studies included. The significance of the effect size was assessed by random‐effects model analysis. Explanation for heterogeneity was explored by meta‐regression by including various moderating factors into the random‐effects model. These included the following: *nesting* – the use of statistical procedures to account for non‐independence of animals from the same litter; *randomization* – the random assignment of animals to each intervention group; *CD:HFD ratio* of macronutrients, fat, carbohydrate (CHO) and protein; *cafeteria diet* – the use of choice diet or supplementation of standard diets with palatable energy‐rich foods; *species*; and *method* by which the outcome was assessed in the studies. Estimates for the SMD and 95% confidence intervals are presented for these models along with the residual heterogeneity unaccounted for in the model (the I^2 beneath each model).

**Figure S4:** Forest plots for male and female leptin. (a) Male offspring, (b) female offspring. In the TOTAL model, estimated SMD and 95% confidence intervals are presented as a summary of all studies. *k* refers to the number of studies included. The significance of the effect size was assessed by random‐effects model analysis. Explanation for heterogeneity was explored by meta‐regression by including various moderating factors into the random‐effects model. These included: *nesting* – the use of statistical procedures to account for non‐independence of animals from the same litter; *randomization* – the random assignment of animals to each intervention group; *CD:HFD ratio* of macronutrients, fat, carbohydrate (CHO) and protein; *cafeteria diet* – the use of choice diet or supplementation of standard diets with palatable energy‐rich foods; *species*; *maternal weight* – an approximation for gestation weight gain taken as the ratio change in weight from pre mating to post lactation. Estimates for the SMD and 95% confidence intervals are presented for these models along with the residual heterogeneity unaccounted for in the model (the I^2 beneath each model).

**Figure S5:** Funnel plots demonstrating publication bias in the metabolic outcomes reported in studies of offspring of mothers maintained on HFD.

**Table S1:** Egger's tests for publication bias

Supporting info item
