# Supplementary material for: Animal models of maternal high fat diet exposure and effects on metabolism in offspring: a meta‐regression analysis
Source: Obes Rev. 2017 Mar 30;18(6):673–86. doi: 10.1111/obr.12524 (PMC5434919; doi:10.1111/obr.12524)
Supplement: Supplementary file 1 — Figure S1: PRISMA chart demonstrating the process for selection of articles. [file OBR-18-673-s002.docx]

Figure S1: PRISMA chart demonstrating the process for selection of articles

Records identified through database searching
(n =5144)

## Screening

## Included

## Eligibility

## Identification

Additional records identified through other sources
(n = 1)

Records after duplicates removed
(n =1844)

Abstracts screened
(n =1844)

Records excluded
(n =1397)

Articles assessed for eligibility
(n =447)

Conference reports
(n =192)

Full text articles assessed for eligibility
(n =255)

Studies included in quantitative synthesis (meta-analysis)
(n = 171)

Articles excluded

Not in English (n=4)

Methods (n=21)

Outcomes not recorded (n=43)

Non-parametric data (n=5)

Insufficient information (n=10)

Unable to obtain paper (n=1)

Total (n=84)
