# Supplementary material for: Animal models of maternal high fat diet exposure and effects on metabolism in offspring: a meta‐regression analysis
Source: Obes Rev. 2017 Mar 30;18(6):673–86. doi: 10.1111/obr.12524 (PMC5434919; doi:10.1111/obr.12524)
Supplement: Supplementary file 2 — Figure S2: Correlations between (a) carbohydrate and (b) fat ratio between intervention and control diets and weaning weight in offspring. Points indicate individual studies. The size of the point is proportional to the inverse square root of the variance in the standardized mean difference of wean weight calculated for each study. A linear model for the fit between the macronutrient ratio and the weaning weight is indicated on each graph. In (a), the correlation between the ratio of carbohydrate content of the diets and wean weight was significant when assessed by Spearman's rank. There was no significant correlation between fat content ratio and wean weight in male offspring. [file OBR-18-673-s003.pptx]

## Slide 1
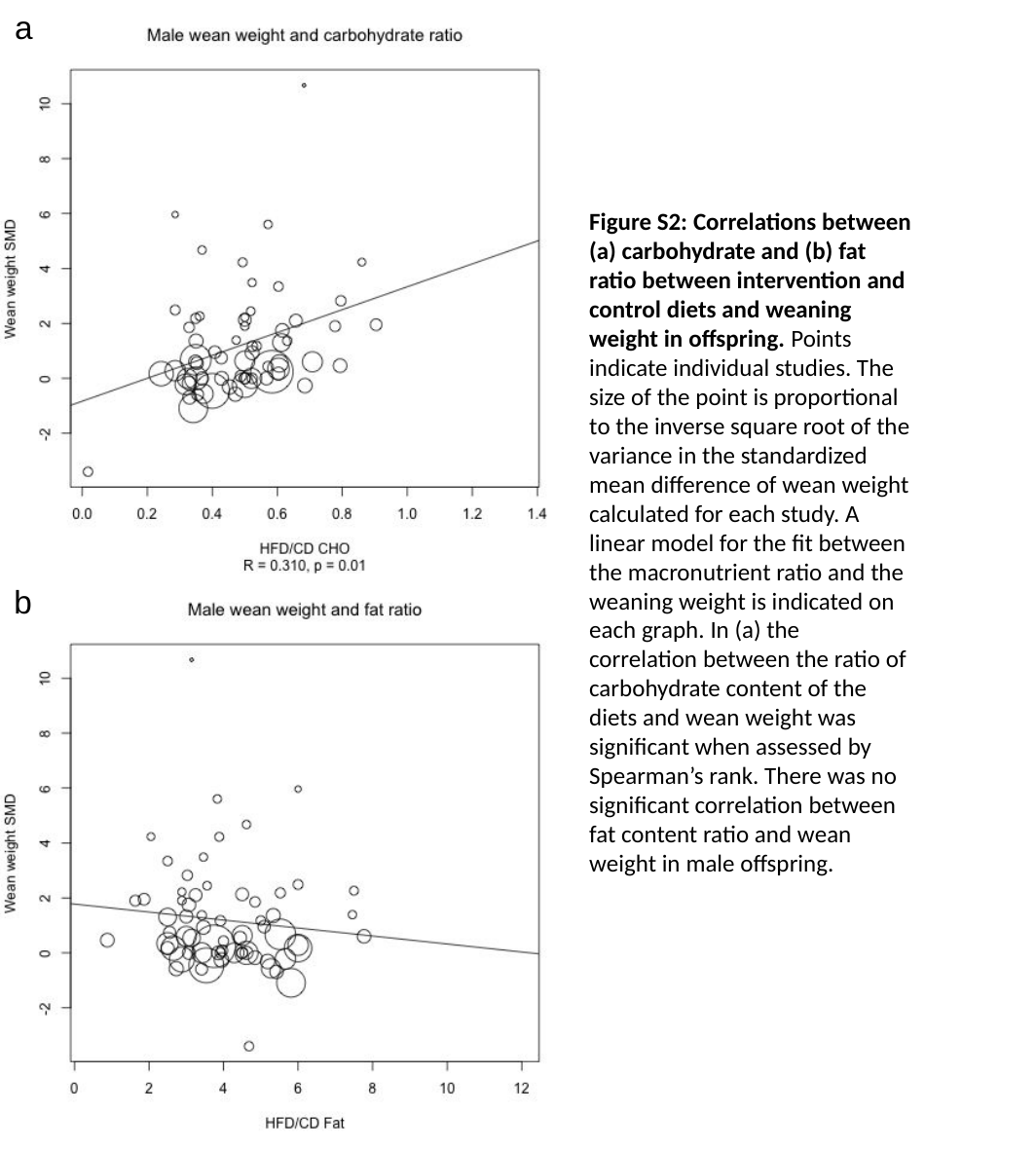

a
Figure S2: Correlations between (a) carbohydrate and (b) fat ratio between intervention and control diets and weaning weight in offspring. Points indicate individual studies. The size of the point is proportional to the inverse square root of the variance in the standardized mean difference of wean weight calculated for each study. A linear model for the fit between the macronutrient ratio and the weaning weight is indicated on each graph. In (a) the correlation between the ratio of carbohydrate content of the diets and wean weight was significant when assessed by Spearman’s rank. There was no significant correlation between fat content ratio and wean weight in male offspring.
b
