# Supplementary material for: Animal models of maternal high fat diet exposure and effects on metabolism in offspring: a meta‐regression analysis
Source: Obes Rev. 2017 Mar 30;18(6):673–86. doi: 10.1111/obr.12524 (PMC5434919; doi:10.1111/obr.12524)
Supplement: Supplementary file 3 — Figure S3: Metabolic outcomes in the offspring of females exposed to HFD: forest plots from studies where male and female data combined. (a) Birthweight, (b) glucose, (c) weaning weight, (d) insulin, (e) final body weight, (f) adiposity, (g) cholesterol, (h) triglycerides, (i) leptin. In the TOTAL model, estimated SMD and 95% confidence intervals are presented as a summary of all studies. k refers to the number of studies included. The significance of the effect size was assessed by random‐effects model analysis. Explanation for heterogeneity was explored by meta‐regression by including various moderating factors into the random‐effects model. These included the following: nesting – the use of statistical procedures to account for non‐independence of animals from the same litter; randomization – the random assignment of animals to each intervention group; CD:HFD ratio of macronutrients, fat, carbohydrate (CHO) and protein; cafeteria diet – the use of choice diet or supplementation of standard diets with palatable energy‐rich foods; species; and method by which the outcome was assessed in the studies. Estimates for the SMD and 95% confidence intervals are presented for these models along with the residual heterogeneity unaccounted for in the model (the I^2 beneath each model). [file OBR-18-673-s004.pptx]

## Slide 1
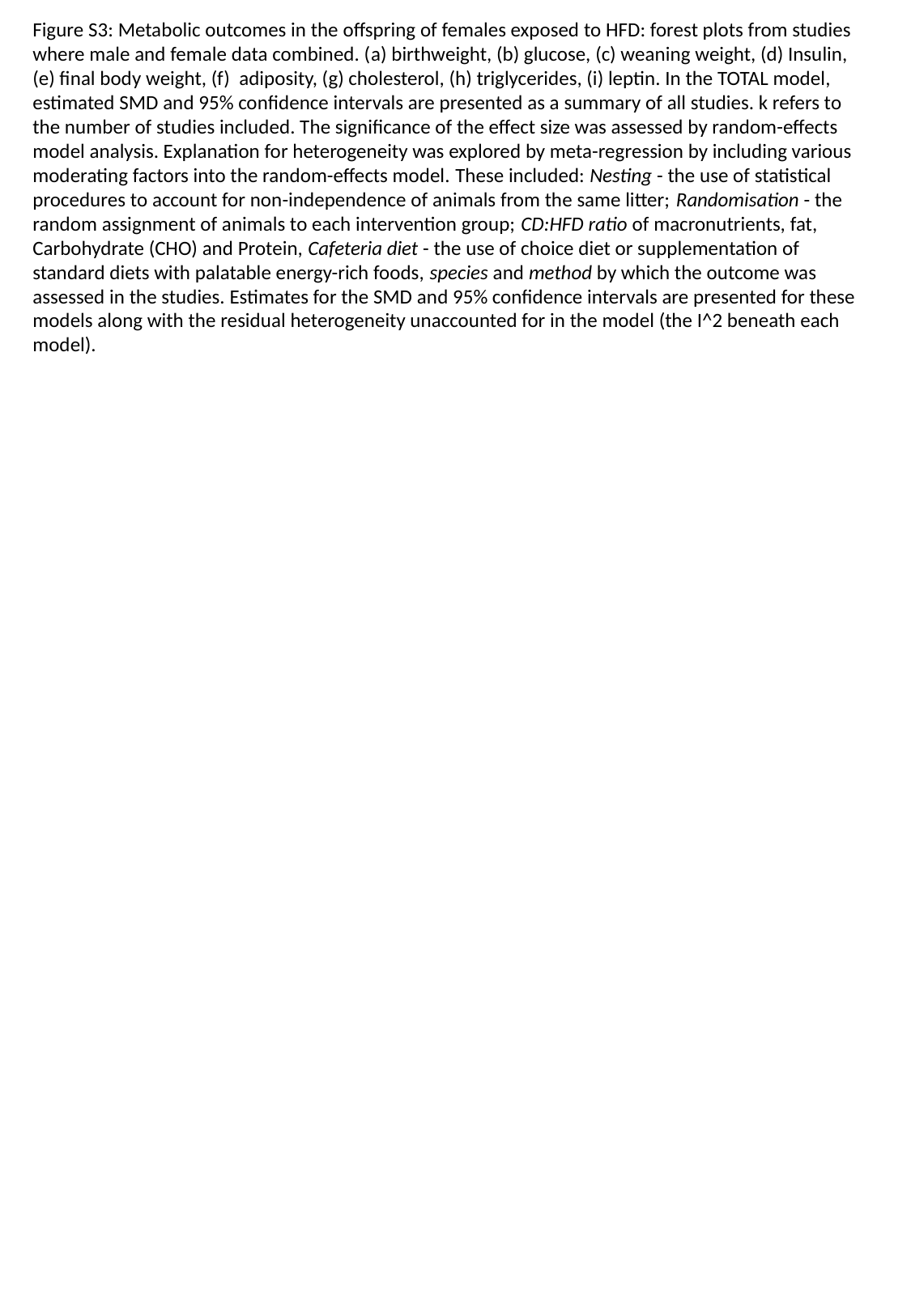

Figure S3: Metabolic outcomes in the offspring of females exposed to HFD: forest plots from studies where male and female data combined. (a) birthweight, (b) glucose, (c) weaning weight, (d) Insulin, (e) final body weight, (f) adiposity, (g) cholesterol, (h) triglycerides, (i) leptin. In the TOTAL model, estimated SMD and 95% confidence intervals are presented as a summary of all studies. k refers to the number of studies included. The significance of the effect size was assessed by random-effects model analysis. Explanation for heterogeneity was explored by meta-regression by including various moderating factors into the random-effects model. These included: Nesting - the use of statistical procedures to account for non-independence of animals from the same litter; Randomisation - the random assignment of animals to each intervention group; CD:HFD ratio of macronutrients, fat, Carbohydrate (CHO) and Protein, Cafeteria diet - the use of choice diet or supplementation of standard diets with palatable energy-rich foods, species and method by which the outcome was assessed in the studies. Estimates for the SMD and 95% confidence intervals are presented for these models along with the residual heterogeneity unaccounted for in the model (the I^2 beneath each model).

## Slide 2
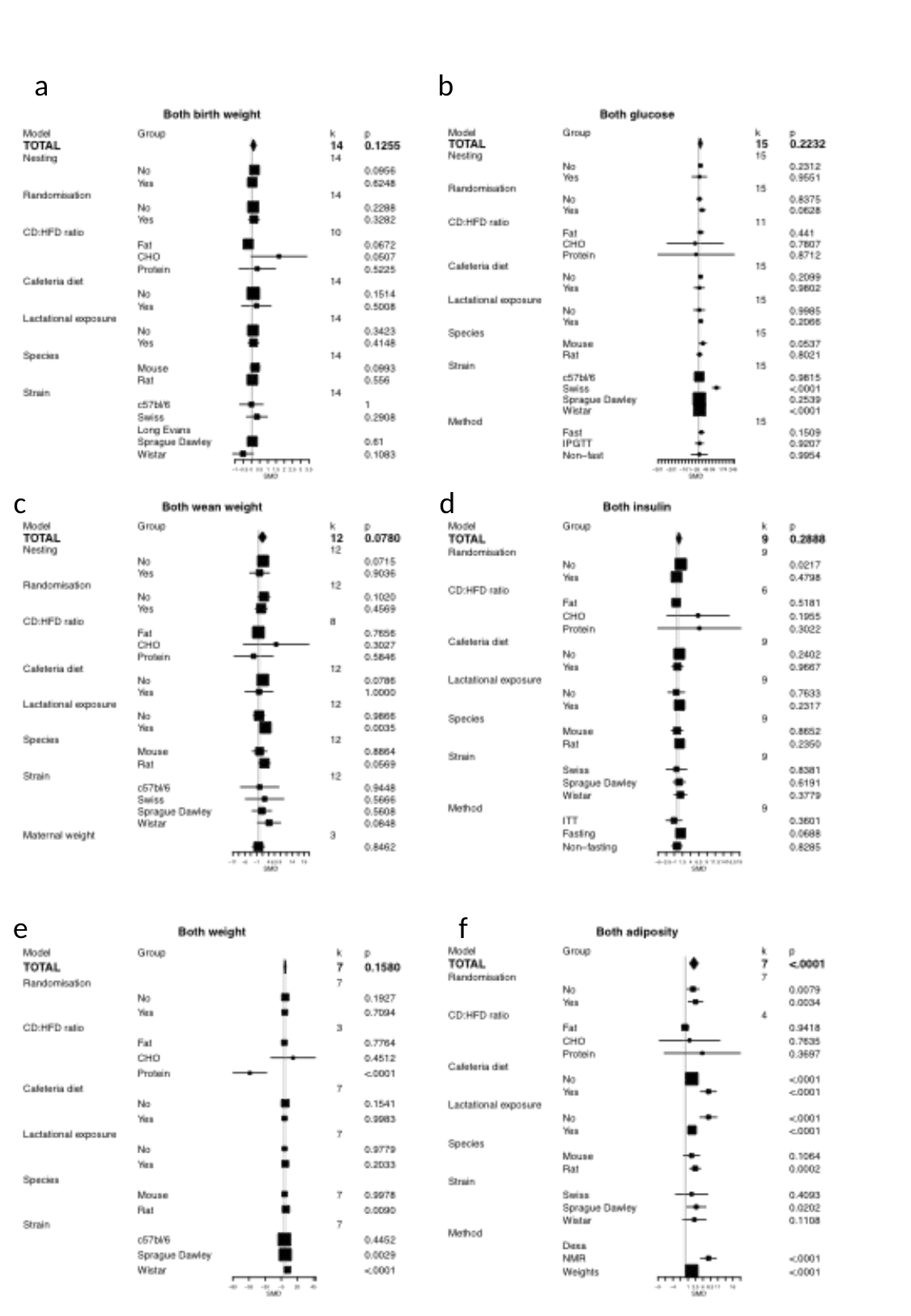

a
b
c
d
e
f

## Slide 3
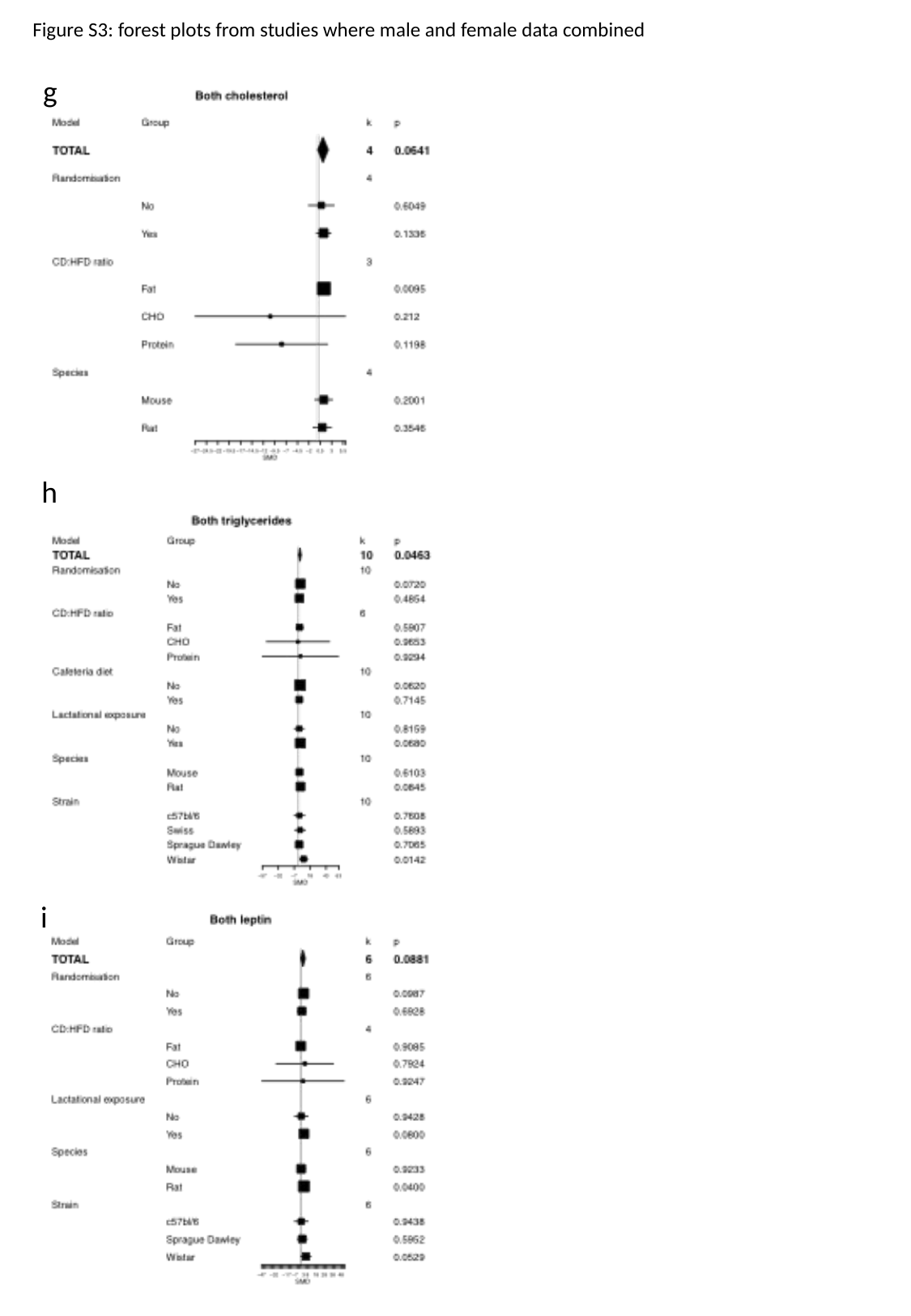

Figure S3: forest plots from studies where male and female data combined
g
h
i
