# Supplementary material for: Animal models of maternal high fat diet exposure and effects on metabolism in offspring: a meta‐regression analysis
Source: Obes Rev. 2017 Mar 30;18(6):673–86. doi: 10.1111/obr.12524 (PMC5434919; doi:10.1111/obr.12524)
Supplement: Supplementary file 5 — Figure S5: Funnel plots demonstrating publication bias in the metabolic outcomes reported in studies of offspring of mothers maintained on HFD. [file OBR-18-673-s006.pptx]

## Slide 1
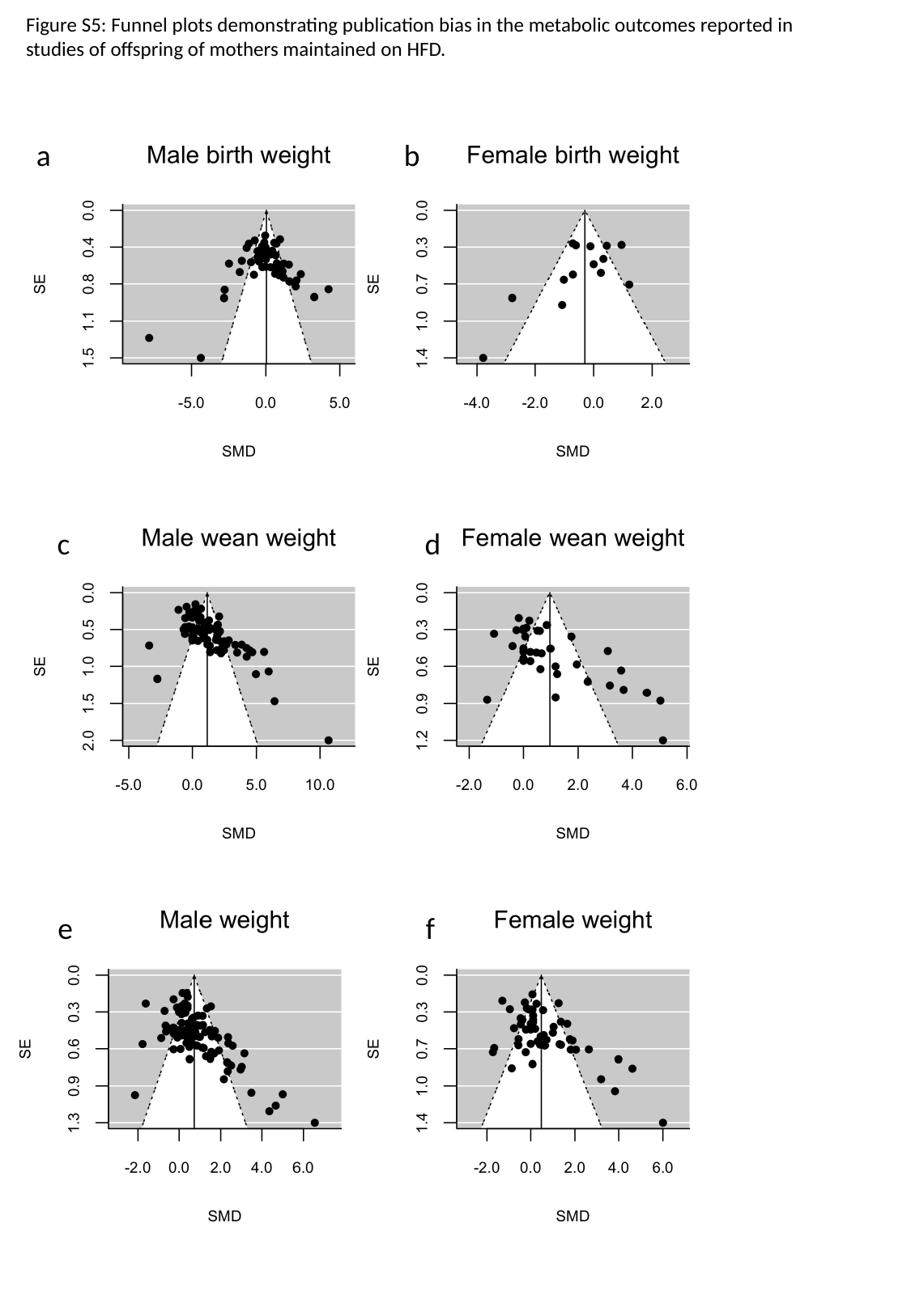

Figure S5: Funnel plots demonstrating publication bias in the metabolic outcomes reported in studies of offspring of mothers maintained on HFD.
a
b
c
d
e
f

## Slide 2
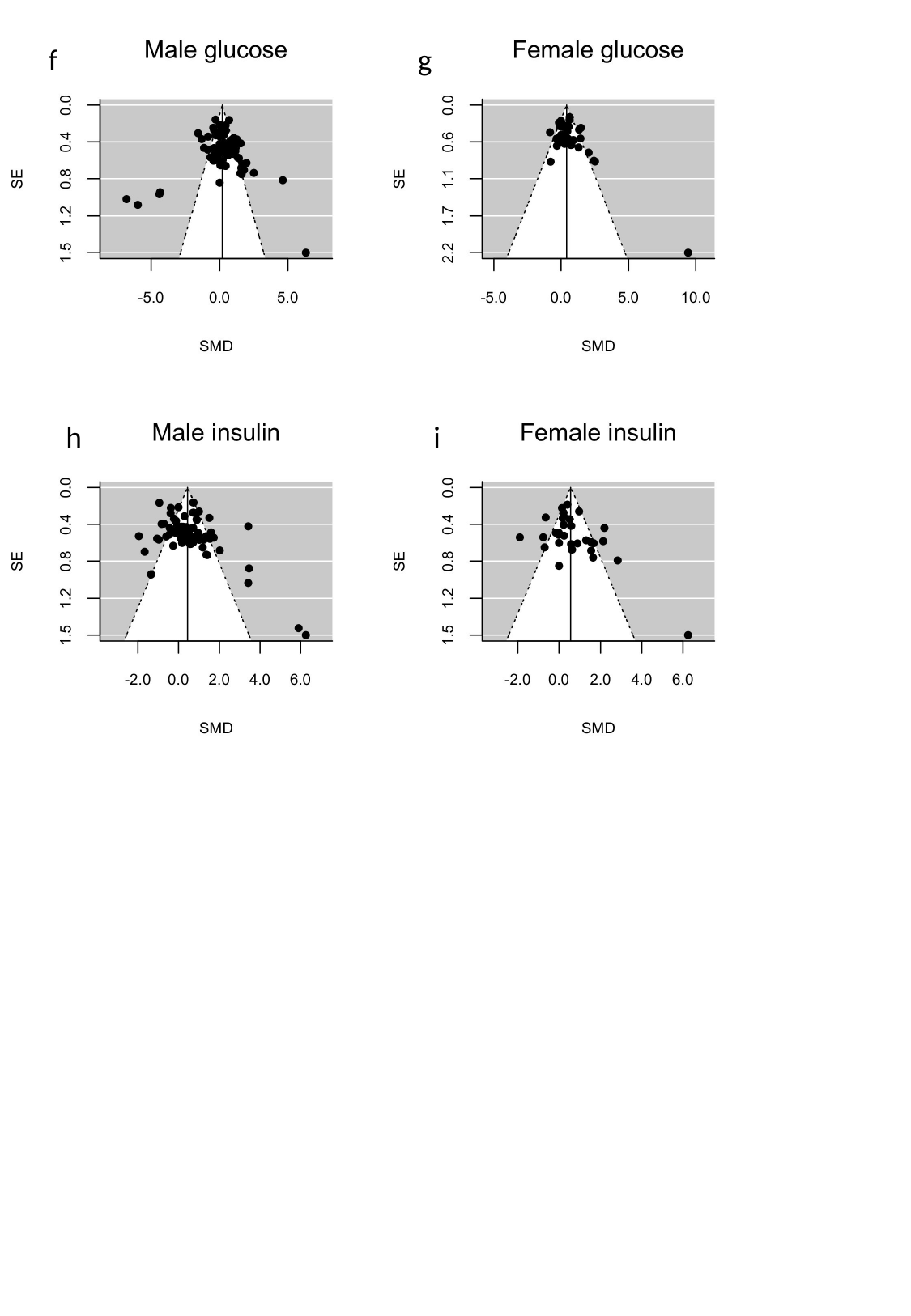

f
g
h
i

## Slide 3
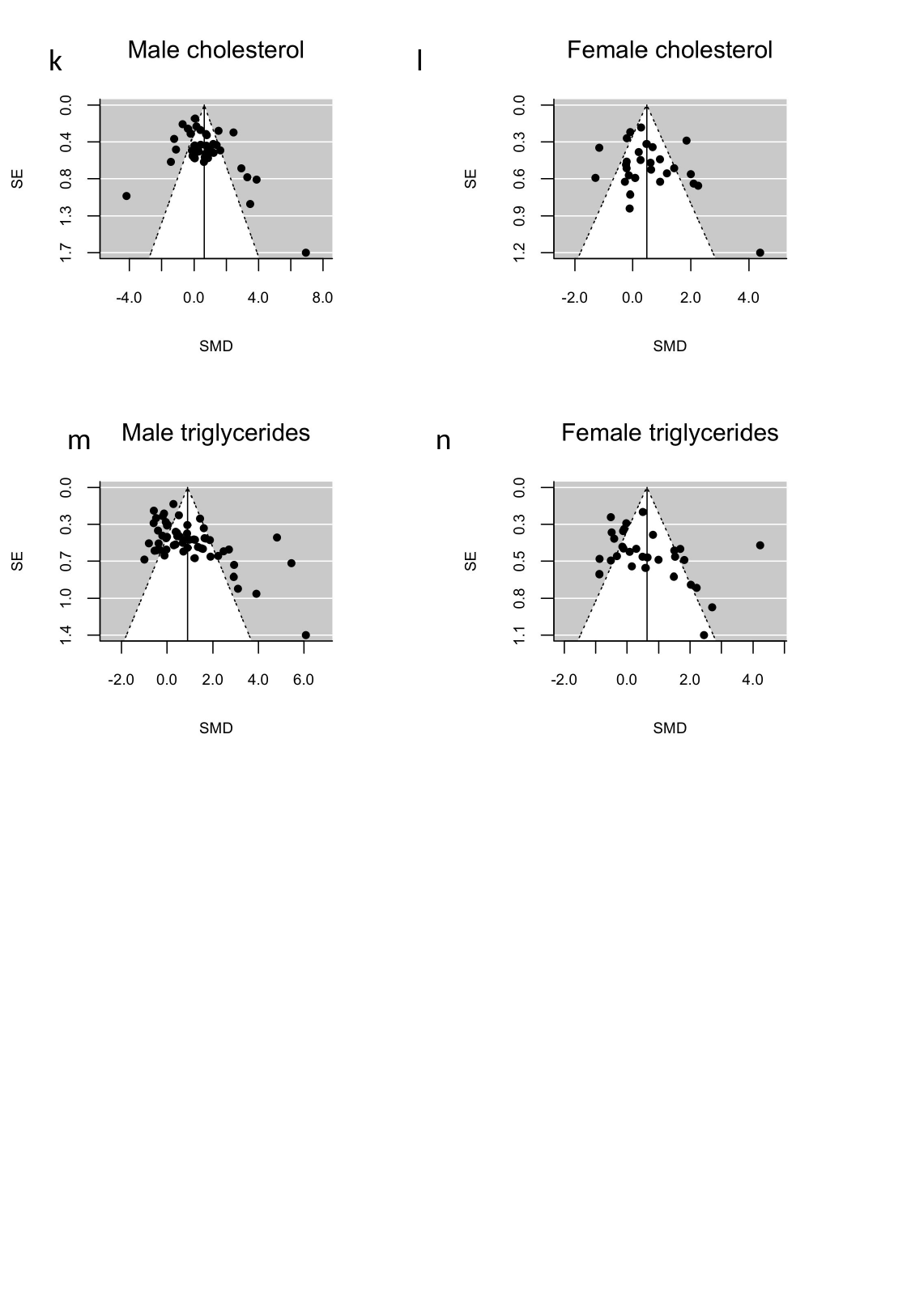

k
l
m
n

## Slide 4
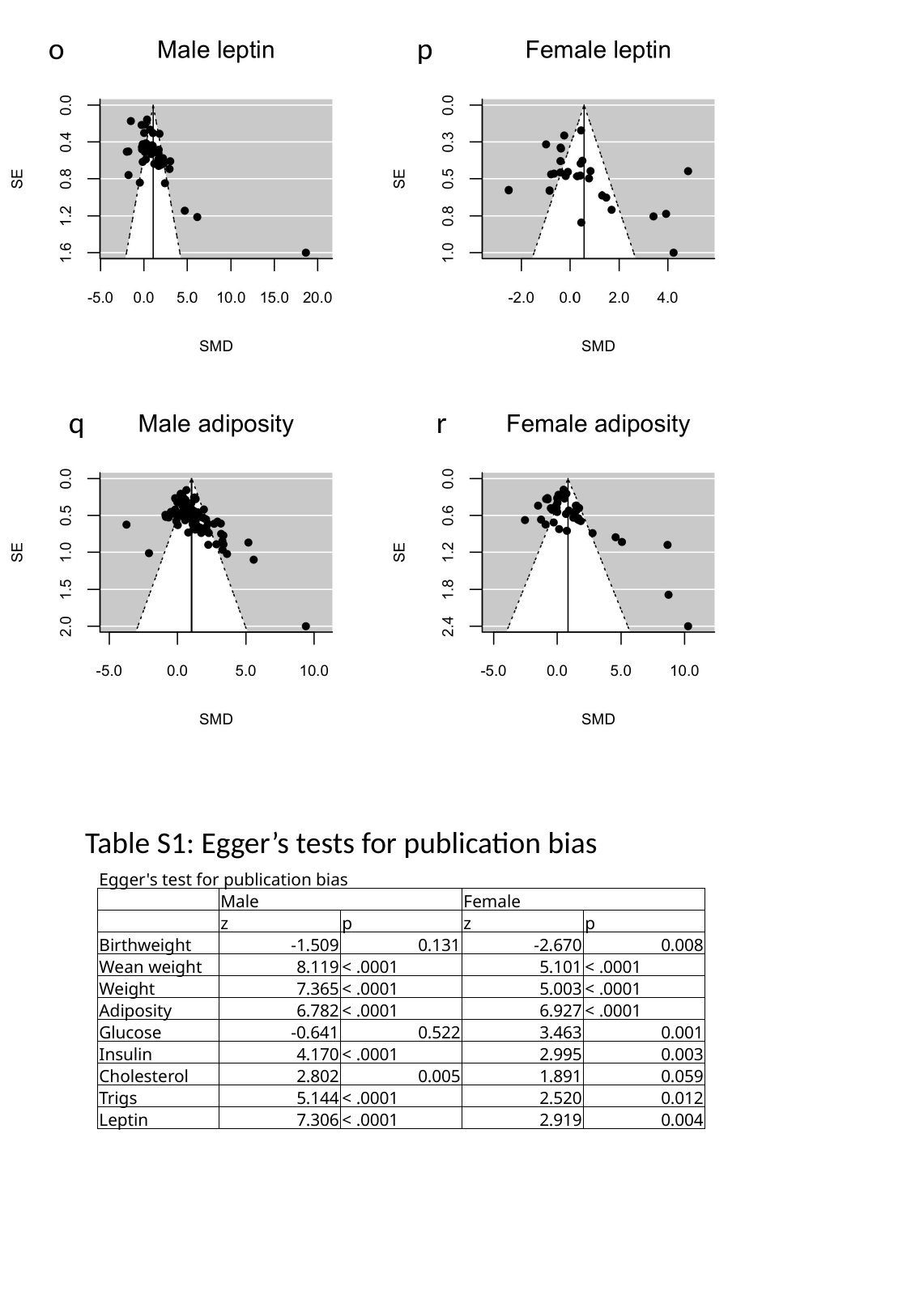

o
p
q
r
Table S1: Egger’s tests for publication bias
| Egger's test for publication bias | | | | |
| --- | --- | --- | --- | --- |
| | Male | | Female | |
| | z | p | z | p |
| Birthweight | -1.509 | 0.131 | -2.670 | 0.008 |
| Wean weight | 8.119 | < .0001 | 5.101 | < .0001 |
| Weight | 7.365 | < .0001 | 5.003 | < .0001 |
| Adiposity | 6.782 | < .0001 | 6.927 | < .0001 |
| Glucose | -0.641 | 0.522 | 3.463 | 0.001 |
| Insulin | 4.170 | < .0001 | 2.995 | 0.003 |
| Cholesterol | 2.802 | 0.005 | 1.891 | 0.059 |
| Trigs | 5.144 | < .0001 | 2.520 | 0.012 |
| Leptin | 7.306 | < .0001 | 2.919 | 0.004 |
