# Supplementary material for: Animal models of maternal high fat diet exposure and effects on metabolism in offspring: a meta‐regression analysis
Source: Obes Rev. 2017 Mar 30;18(6):673–86. doi: 10.1111/obr.12524 (PMC5434919; doi:10.1111/obr.12524)
Supplement: Supplementary file 6 — Table S1: Egger's tests for publication bias [file OBR-18-673-s007.pdf]

Animal models of maternal high fat diet exposure and effects on metabolism in offspring: A meta-regression analysis.

Ribaroff GA, Wastnedge E, Drake AJ, Sharpe RM, Chambers TJG.

Supplementary figures and table

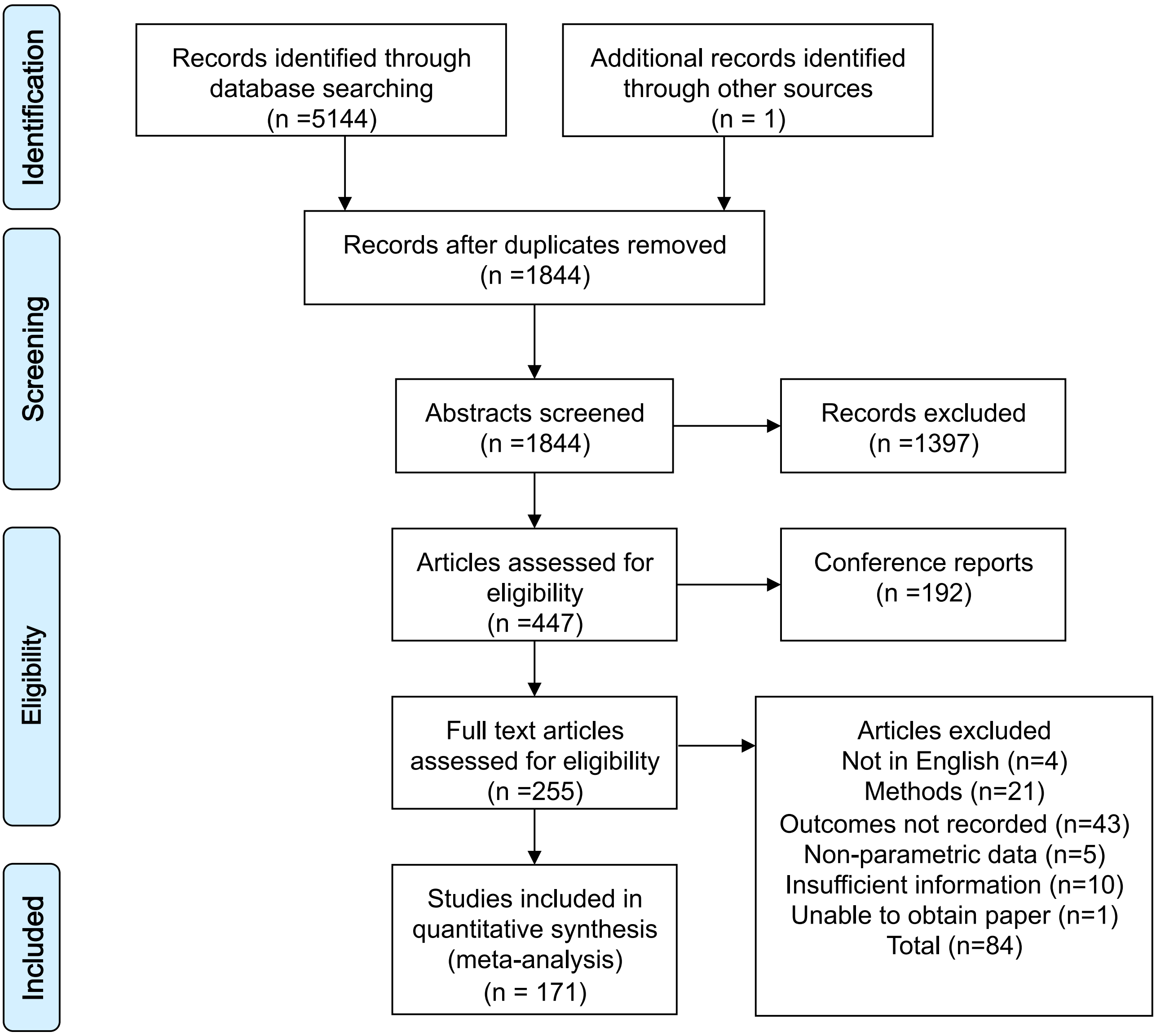

Figure S1: PRISMA chart demonstrating the process for selection of articles

a

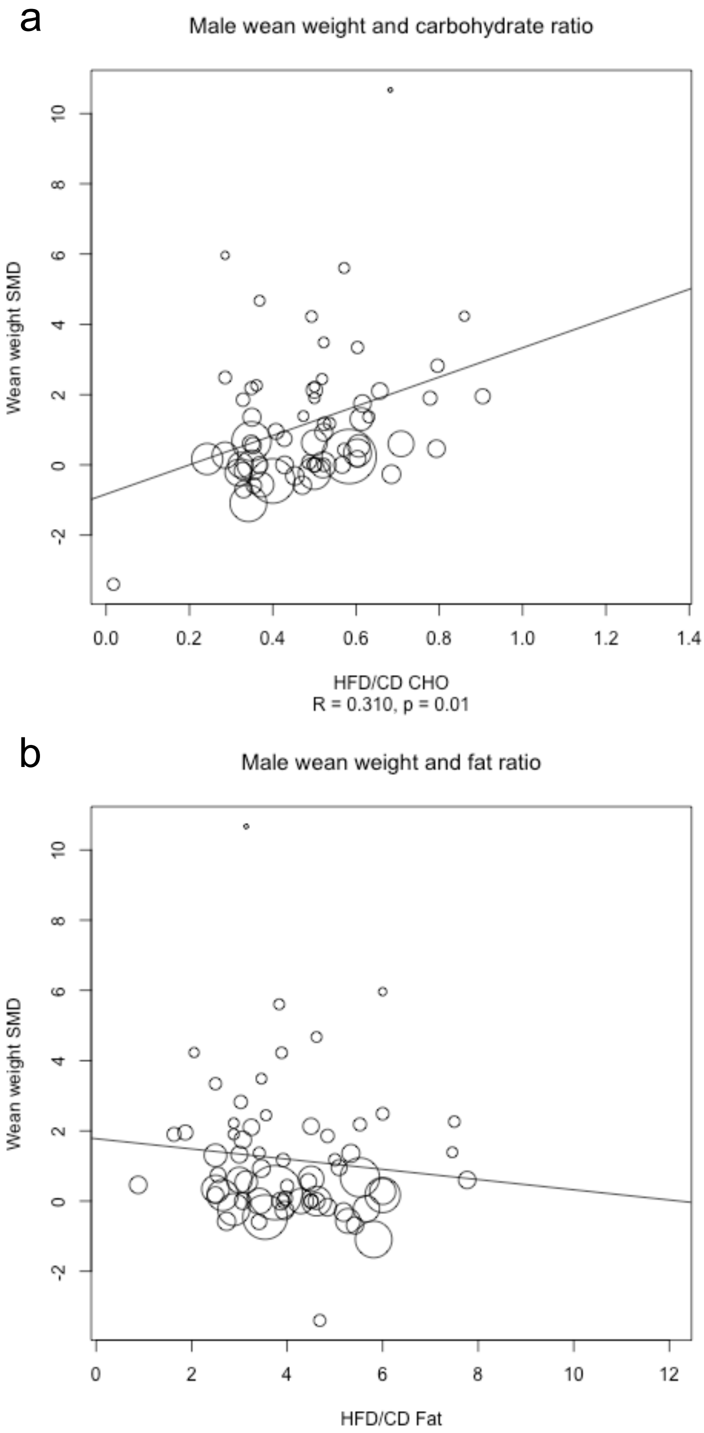

Figure S2: Correlations between (a) carbohydrate and (b) fat ratio between intervention and control diets and weaning weight in offspring. Points indicate individual studies. The size of the point is proportional to the inverse square root of the variance in the standardized mean difference of wean weight calculated for each study. A linear model for the fit between the macronutrient ratio and the weaning weight is indicated on each graph. In (a) the correlation between the ratio of carbohydrate content of the diets and wean weight was significant when assessed by Spearman's rank. There was no significant correlation between fat content ratio and wean weight in male offspring.

Figure S3: Metabolic outcomes in the offspring of females exposed to HFD: forest plots from studies where male and female data combined. (a) birthweight, (b) glucose, (c) weaning weight, (d) Insulin, (e) final body weight, (f) adiposity, (g) cholesterol, (h) triglycerides, (i) leptin. In the TOTAL model, estimated SMD and 95% confidence intervals are presented as a summary of all studies. k refers to the number of studies included. The significance of the effect size was assessed by random-effects model analysis. Explanation for heterogeneity was explored by meta-regression by including various moderating factors into the random-effects model. These included: *Nesting* - the use of statistical procedures to account for non-independence of animals from the same litter; *Randomisation* - the random assignment of animals to each intervention group; *CD:HFD ratio* of macronutrients, fat, Carbohydrate (CHO) and Protein, *Cafeteria diet* - the use of choice diet or supplementation of standard diets with palatable energy-rich foods, *species* and *method* by which the outcome was assessed in the studies. Estimates for the SMD and 95% confidence intervals are presented for these models along with the residual heterogeneity unaccounted for in the model (the I^2 beneath each model).

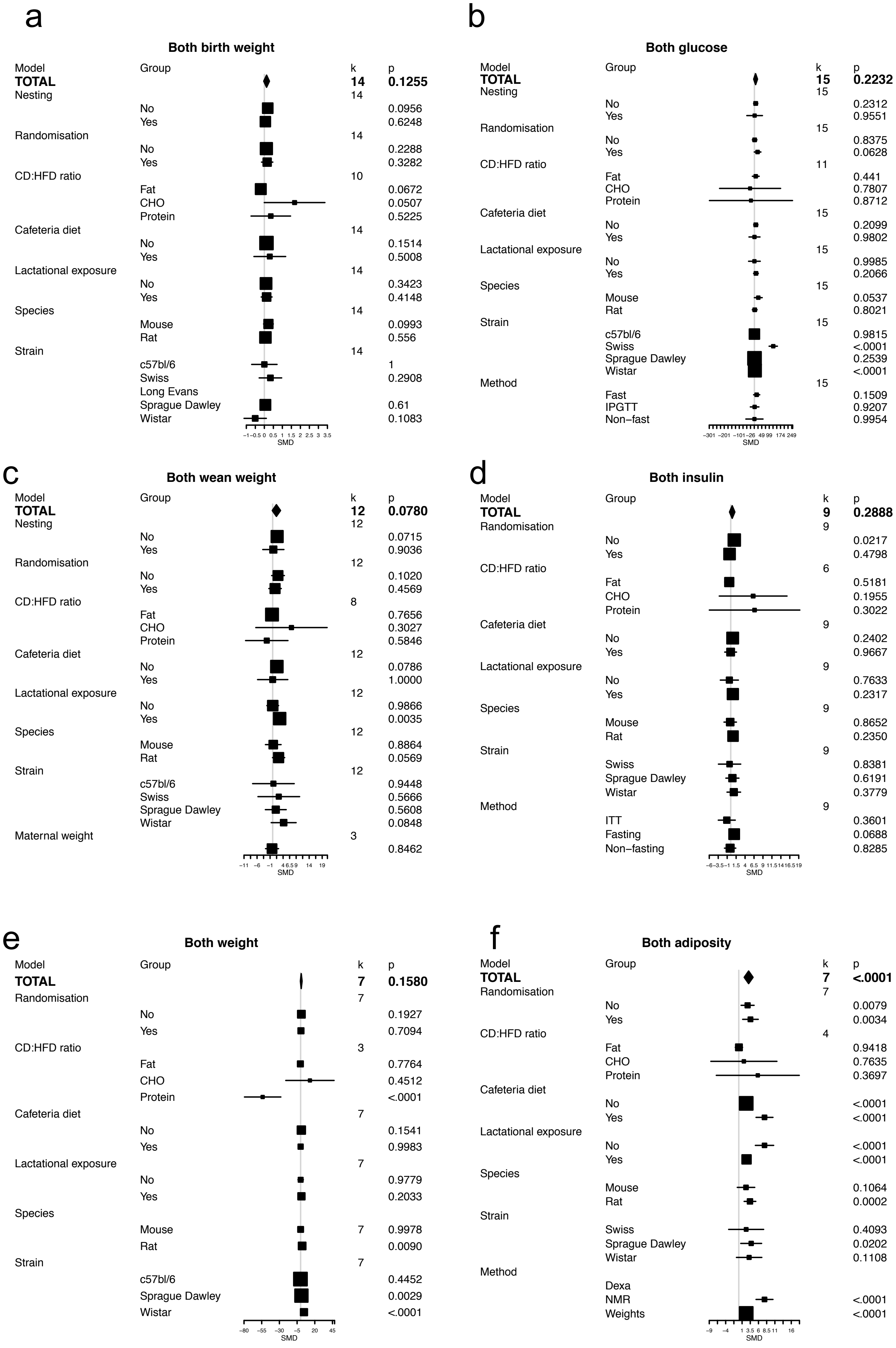
a
Both birth weight
Model
Group
k
p
TOTAL
14
0.1255
Nesting
14
No
0.0956
Yes
0.6248
Randomisation
14
No
0.2288
Yes
0.3282
CD:HFD ratio
10
Fat
0.0672
CHO
0.0507
Protein
0.5225
Cafeteria diet
14
No
0.1514
Yes
0.5008
Lactational exposure
14
No
0.3423
Yes
0.4148
Species
14
Mouse
0.0993
Rat
0.556
Strain
14
c57bl/6
1
Swiss
0.2908
Long Evans
Sprague Dawley
0.61
Wistar
0.1083
SMD
b
Both glucose
Model
Group
k
p
TOTAL
15
0.2232
Nesting
15
No
0.2312
Yes
0.9551
Randomisation
15
No
0.8375
Yes
0.0628
CD:HFD ratio
11
Fat
0.441
CHO
0.7807
Protein
0.8712
Cafeteria diet
15
No
0.2099
Yes
0.9802
Lactational exposure
15
No
0.9985
Yes
0.2066
Species
15
Mouse
0.0537
Rat
0.8021
Strain
15
c57bl/6
0.9815
Swiss
<.0001
Sprague Dawley
0.2539
Wistar
<.0001
Method
15
Fast
0.1509
IPGTT
0.9207
Non−fast
0.9954
SMD
c
Both wean weight
Model
Group
k
p
TOTAL
12
0.0780
Nesting
12
No
0.0715
Yes
0.9036
Randomisation
12
No
0.1020
Yes
0.4569
CD:HFD ratio
8
Fat
0.7656
CHO
0.3027
Protein
0.5846
Cafeteria diet
12
No
0.0786
Yes
1.0000
Lactational exposure
12
No
0.9866
Yes
0.0035
Species
12
Mouse
0.8864
Rat
0.0569
Strain
12
c57bl/6
0.9448
Swiss
0.5666
Sprague Dawley
0.5608
Wistar
0.0848
Maternal weight
3
0.8462
SMD
d
Both insulin
Model
Group
k
p
TOTAL
9
0.2888
Randomisation
9
No
0.0217
Yes
0.4798
CD:HFD ratio
6
Fat
0.5181
CHO
0.1955
Protein
0.3022
Cafeteria diet
9
No
0.2402
Yes
0.9667
Lactational exposure
9
No
0.7633
Yes
0.2317
Species
9
Mouse
0.8652
Rat
0.2350
Strain
9
Swiss
0.8381
Sprague Dawley
0.6191
Wistar
0.3779
Method
9
ITT
0.3601
Fasting
0.0688
Non−fasting
0.8285
SMD
e
Both weight
Model
Group
k
p
TOTAL
7
0.1580
Randomisation
7
No
0.1927
Yes
0.7094
CD:HFD ratio
3
Fat
0.7764
CHO
0.4512
Protein
<.0001
Cafeteria diet
7
No
0.1541
Yes
0.9983
Lactational exposure
7
No
0.9779
Yes
0.2033
Species
Mouse
7
0.9978
Rat
0.0090
Strain
7
c57bl/6
0.4452
Sprague Dawley
0.0029
Wistar
<.0001
SMD
f
Both adiposity
Model
Group
k
p
TOTAL
7
<.0001
Randomisation
7
No
0.0079
Yes
0.0034
CD:HFD ratio
4
Fat
0.9418
CHO
0.7635
Protein
0.3697
Cafeteria diet
No
<.0001
Yes
<.0001
Lactational exposure
No
<.0001
Yes
<.0001
Species
Mouse
0.1064
Rat
0.0002
Strain
Swiss
0.4093
Sprague Dawley
0.0202
Wistar
0.1108
Method
Dexa
NMR
<.0001
Weights
<.0001
SMD

Figure S3: forest plots from studies where male and female data combined

g

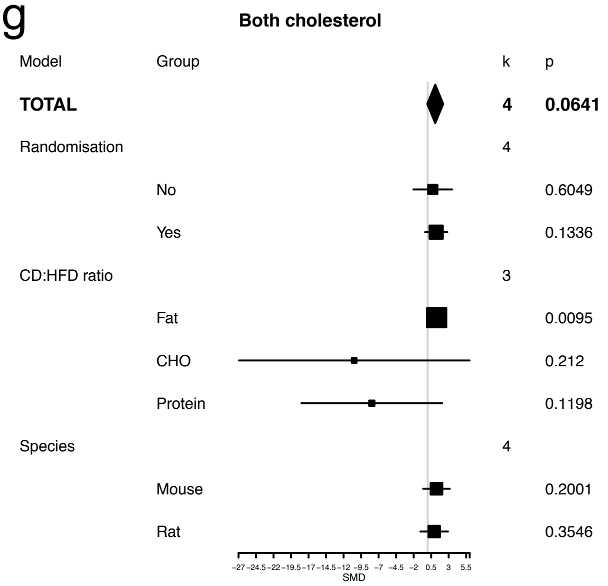

h

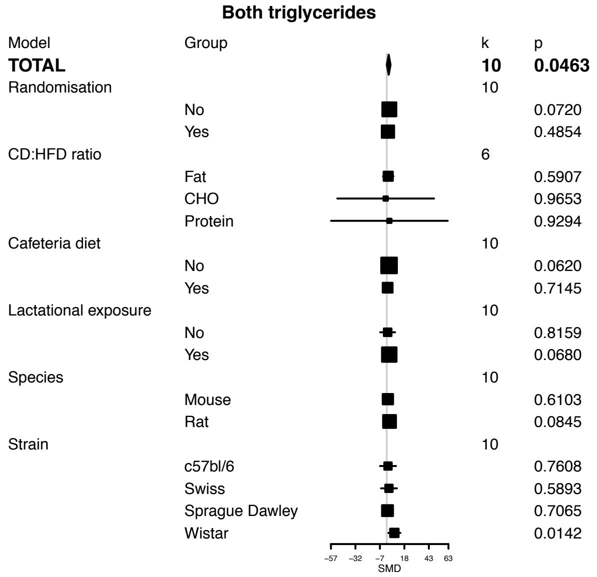

i

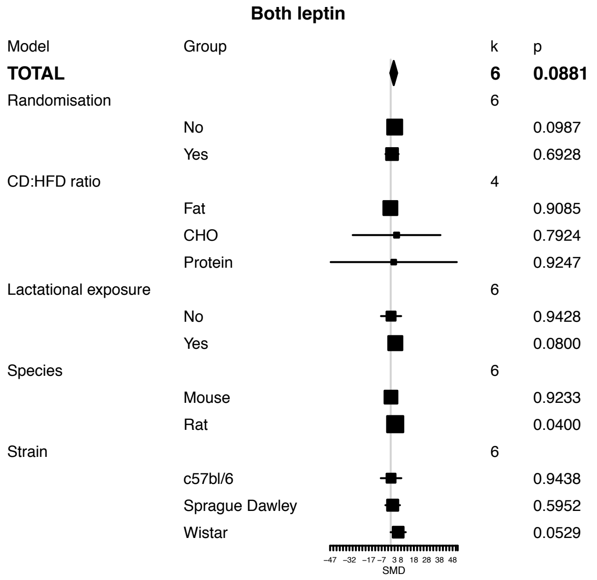

Figure S4: Forest plots for male and female leptin. (a) Male offspring, (b) female offspring. In the TOTAL model, estimated SMD and 95% confidence intervals are presented as a summary of all studies. k refers to the number of studies included. The significance of the effect size was assessed by random-effects model analysis. Explanation for heterogeneity was explored by meta-regression by including various moderating factors into the random-effects model. These included: *Nesting* - the use of statistical procedures to account for non-independence of animals from the same litter; *Randomisation* - the random assignment of animals to each intervention group; *CD:HFD ratio* of macronutrients, fat, Carbohydrate (CHO) and Protein, *Cafeteria diet* - the use of choice diet or supplementation of standard diets with palatable energy-rich foods, *species*, *maternal weight* - an approximation for gestation weight gain taken as the ratio change in weight from pre mating to post lactation. Estimates for the SMD and 95% confidence intervals are presented for these models along with the residual heterogeneity unaccounted for in the model (the I^2 beneath each model).

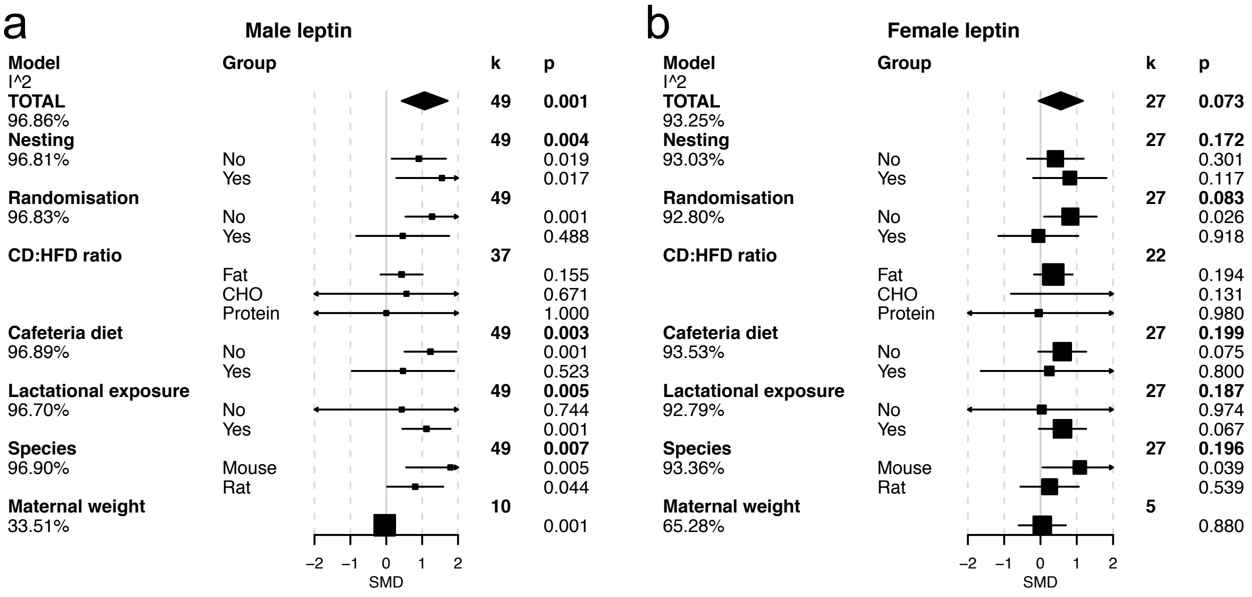

Figure S5: Funnel plots demonstrating publication bias in the metabolic outcomes reported in studies of offspring of mothers maintained on HFD.

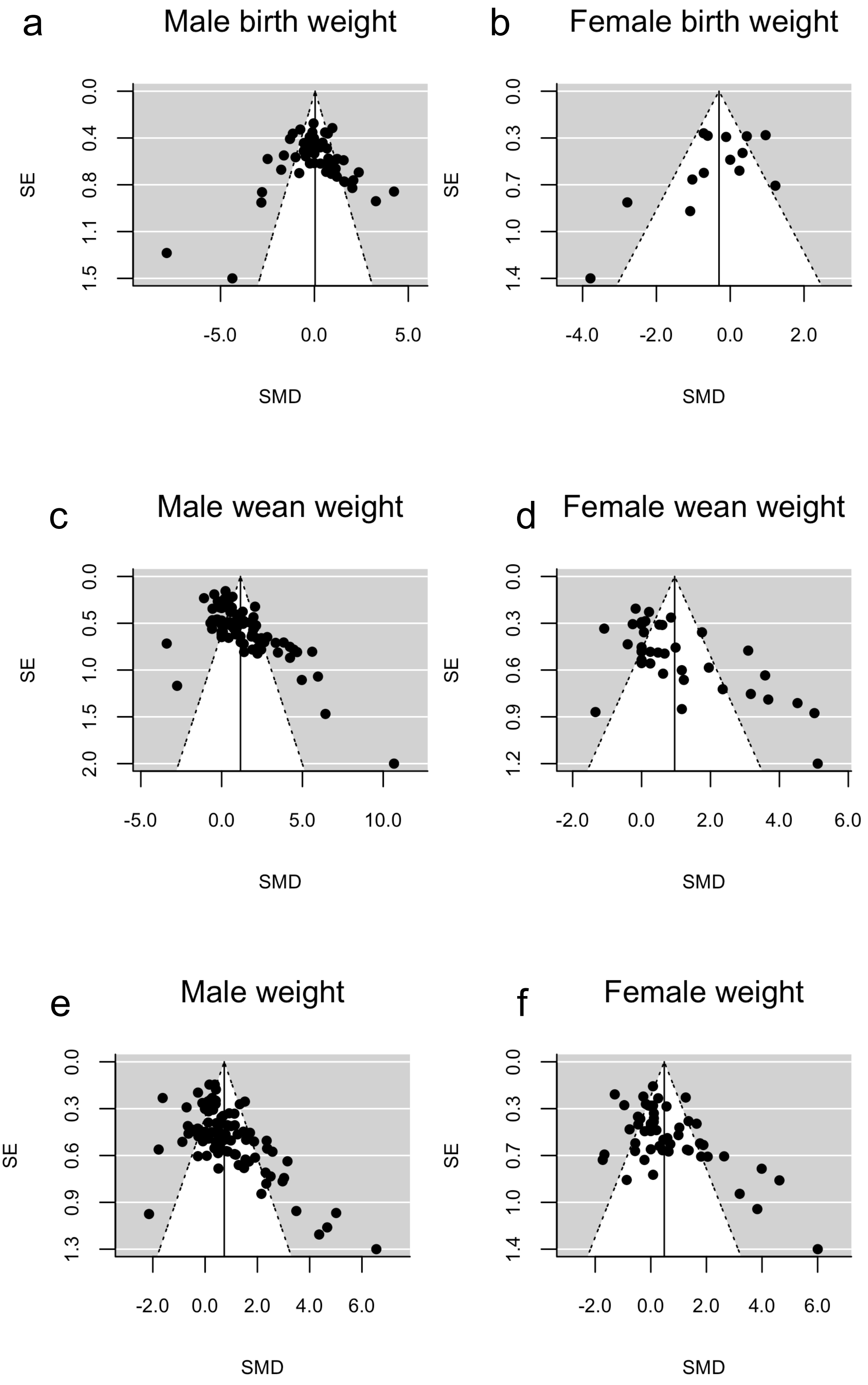

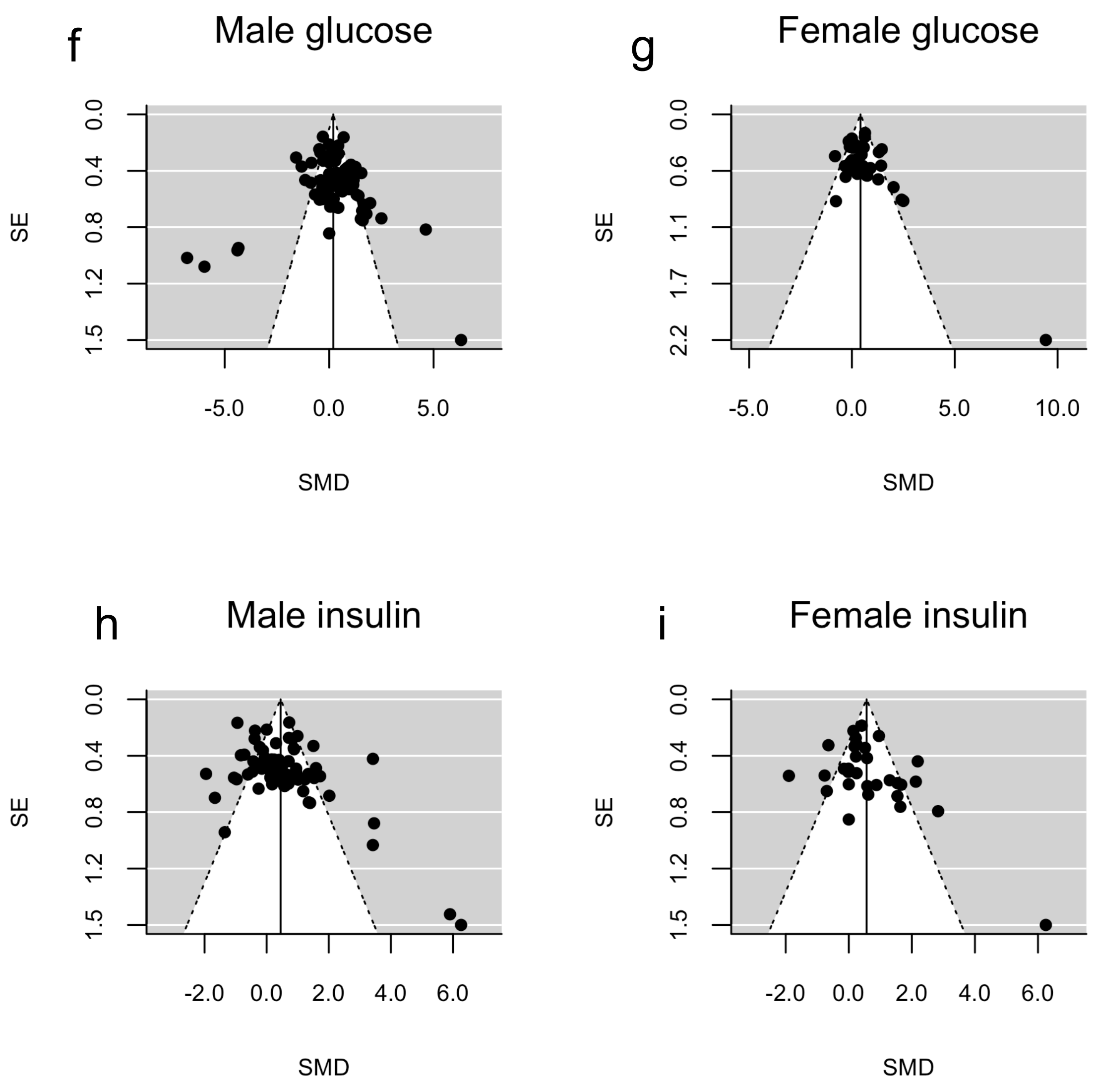
f
Male glucose
SE
0.0
0.4
0.8
1.2
1.5
-5.0
0.0
5.0
SMD
g
Female glucose
SE
0.0
0.6
1.1
1.7
2.2
-5.0
0.0
5.0
10.0
SMD
h
Male insulin
SE
0.0
0.4
0.8
1.2
1.5
-2.0
0.0
2.0
4.0
6.0
SMD
i
Female insulin
SE
0.0
0.4
0.8
1.2
1.5
-2.0
0.0
2.0
4.0
6.0
SMD

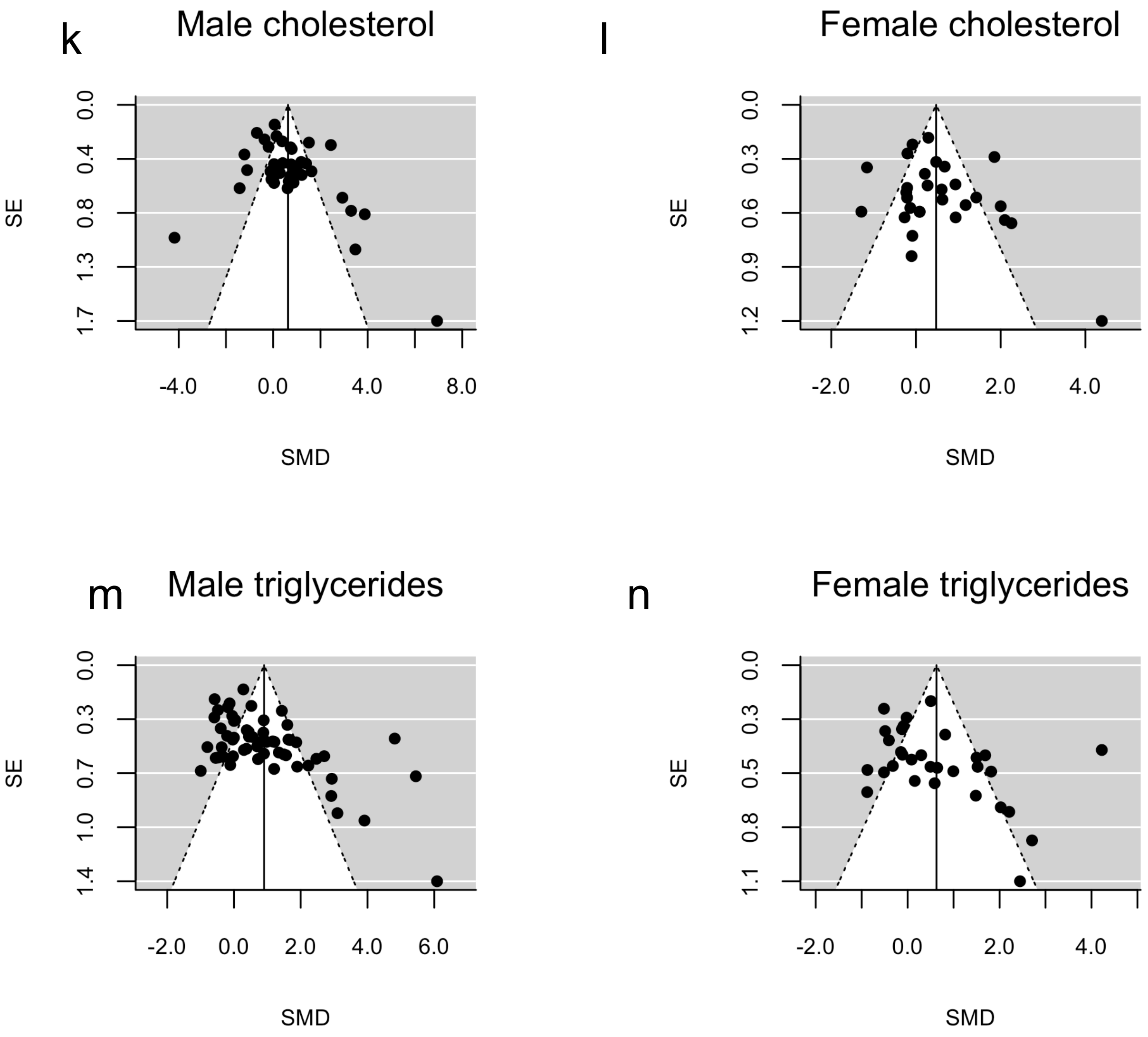
k
Male cholesterol
SE
0.0
0.4
0.8
1.3
1.7
-4.0
0.0
4.0
8.0
SMD
l
Female cholesterol
SE
0.0
0.3
0.6
0.9
1.2
-2.0
0.0
2.0
4.0
SMD
m
Male triglycerides
SE
0.0
0.3
0.7
1.0
1.4
-2.0
0.0
2.0
4.0
6.0
SMD
n
Female triglycerides
SE
0.0
0.3
0.5
0.8
1.1
-2.0
0.0
2.0
4.0
SMD

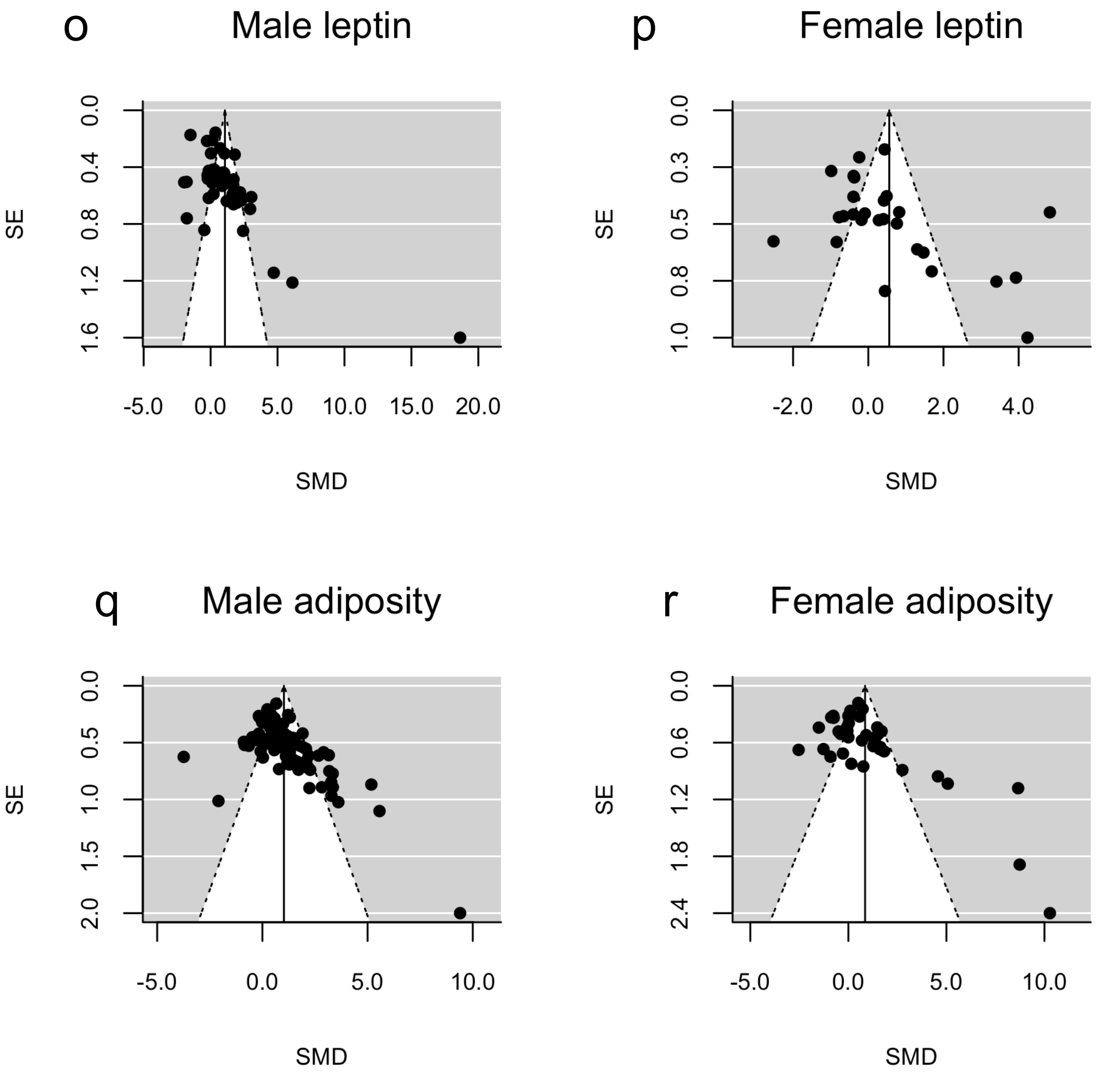

Table S1: Egger's tests for publication bias

Egger's test for publication bias

	Male		Female	
	z	p	z	p
Birthweight	-1.509	0.131	-2.670	**0.008**
Wean weight	8.119	**< .0001**	5.101	**< .0001**
Weight	7.365	**< .0001**	5.003	**< .0001**
Adiposity	6.782	**< .0001**	6.927	**< .0001**
Glucose	-0.641	0.522	3.463	**0.001**
Insulin	4.170	**< .0001**	2.995	**0.003**
Cholesterol	2.802	**0.005**	1.891	0.059
Trigs	5.144	**< .0001**	2.520	**0.012**
Leptin	7.306	**< .0001**	2.919	**0.004**
